# HERVK-mediated regulation of neighboring genes: implications for breast cancer prognosis

**DOI:** 10.1186/s12977-024-00636-z

**Published:** 2024-02-22

**Authors:** Boying Liang, Tengyue Yan, Huilin Wei, Die Zhang, Lanxiang Li, Zengjing Liu, Wen Li, Yuluan Zhang, Nili Jiang, Qiuxia Meng, Guiyang Jiang, Yanling Hu, Jing Leng

**Affiliations:** 1 Department of Immunology, School of Basic Medical Sciences, Guangxi Medical University, Nanning, 530021 Guangxi China; 2 Collaborative Innovation Centre of Regenerative Medicine and Medical Bioresource Development and Application Co-Constructed by the Province and Ministry, Guangxi Medical University, Nanning, China; 3 School of Institute of Life Sciences, Guangxi Medical University, Nanning, China; 4 Genomic Experimental Center, Guangxi Medical University, Nanning, China; 5 Guangxi Key Laboratory of Translational Medicine for Treating High-Incidence Infectious Diseases with Integrative Medicine, Nanning, China

**Keywords:** Human endogenous retrovirus-K, Breast cancer, Neighborhood gene, Immune infiltration, Prognosis

## Abstract

Human endogenous retroviruses (HERVs) are the remnants of ancient retroviral infections integrated into the human genome. Although most HERVs are silenced or rendered inactive by various regulatory mechanisms, they retain the potential to influence the nearby genes. We analyzed the regulatory map of 91 HERV-Ks on neighboring genes in human breast cancer and investigated the impact of HERV-Ks on the tumor microenvironment (TME) and prognosis of breast cancer. Nine RNA-seq datasets were obtained from GEO and NCBI SRA. Differentially expressed genes and HERV-Ks were analyzed using DESeq2. Validation of high-risk prognostic candidate genes using TCGA data. These included Overall survival (multivariate Cox regression model), immune infiltration analysis (TIMER), tumor mutation burden (maftools), and drug sensitivity analysis (GSCA). A total of 88 candidate genes related to breast cancer prognosis were screened, of which CD48, SLAMF7, SLAMF1, IGLL1, IGHA1, and LRRC8A were key genes. Functionally, these six key genes were significantly enriched in some immune function-related pathways, which may be associated with poor prognosis for breast cancer (p = 0.00016), and the expression levels of these genes were significantly correlated with the sensitivity of breast cancer treatment-related drugs. Mechanistically, they may influence breast cancer development by modulating the infiltration of various immune cells into the TME. We further experimentally validated these genes to confirm the results obtained from bioinformatics analysis. This study represents the first report on the regulatory potential of HERV-K in the neighboring breast cancer genome. We identified three key HERV-Ks and five neighboring genes that hold promise as novel targets for future interventions and treatments for breast cancer.

**Supplementary Information:**

The online version contains supplementary material available at 10.1186/s12977-024-00636-z.

## Introduction

Breast cancer is widespread worldwide and poses a major threat to women's health. Breast cancer in women is estimated to surpass lung cancer by 2020 to become the most diagnosed cancer for the first time [1]. Breast cancer accounted for the highest percentage of cancer-related deaths among women in 2020 and was the second leading cause of cancer-related deaths in women [2, 3]. The genetic and phenotypic heterogeneity of breast cancer and the lack of clear molecular targets leading to its early detection, diagnosis, and treatment remain great challenges. Multiple HERV-K transcripts and protein overexpression have been reported in tumor specimens from patients with breast cancer patients [4–6]. Increasing scientific evidence has demonstrated that aberrant activation of HERV-K has a significant impact on the diagnosis and treatment of human cancers [7–10].

HERVs are important components of the host's evolutionary process. With the accumulation of mutations in the HERV sequence, most of the HERV lost its coding ability, but it was still found that its promoter activity was not destroyed [11]. Different types of LTRs (5' pre-viral, 3' pre-viral, and solo) as well as their relative positions to genes, may influence the activity and regulatory characteristics of LTR promoters [12, 13]. HERVs may mediate tumor occurrence through chromosomal rearrangements mediated by homologous recombination, encoding oncogenic proteins, and mediating immune suppression. Additionally, the regulation of neighboring gene expression by HERvs may be an important pathophysiological mechanism [14, 15]. The distribution of HERVs in human genes also provides favorable conditions for the regulation of neighboring gene expression [16].

The HERV-K family is considered the most biologically active member of the HERV family, and its abnormal expression and activation in human tumors and other diseases have been extensively studied [17]. The complete HERV-K sequence is approximately 9.5 kb long and includes four viral protein-coding sequences (gag, pro, pol, and env) located between two identical long terminal repeat (LTR) sequences [18, 19]. The Flanker virus LTR (Long Terminal Repeat) is a critical component of the viral genome that contains U3R and U5 regions arranged in a 5' to 3' direction. These regions are essential for the replication and transcription of the viral genome. Several important functional elements within the LTR play vital roles in regulating viral gene expression and replication, including promoters, enhancers, TATA-independent polyadenylation sites, and multiple transcription factor-binding sites (TFBSs) [20]. The regulatory effects of HERV-K depend on the integration site and surrounding genes [21, 22]. Based on phylogenetic analyses, the LTR sequences of HML-2 have been classified into three subgroups: LTR5A, LTR5B, and LTR5Hs [16]. Highly active LTR5Hs/LTR5 can act as distal enhancers to regulate host genes [12]. Certain HERV-K sequences have been shown to specifically regulate specific genes or genomic regions. HERV-K-mediated non-allelic gene recombination induces chromosomal rearrangements in prostate cancer. The upstream sequence of HERV-K 5'LTR, situated at 22q11.23, can undergo recombination with the transcription factor ETS, resulting in the upregulation of the proto-oncogene ETV1 (ETS variant 1). This process may promote cancer development and progression [23].

Combining transcriptomic and clinical data to determine prognostic indicators for breast cancer can enhance predictive accuracy to some extent and has important clinical implications. The tumor microenvironment (TME) refers to the noncancerous cells and components present within a tumor and the molecules they produce and release. Continuous interactions between tumor cells and the tumor microenvironment (TME) are closely linked to tumors' occurrence, progression, and metastasis [24, 25]. In this study, we performed a joint analysis of GEO and TCGA data, validated candidate genes associated with breast cancer prognosis selected from GSE using TCGA data, and explored patient prognosis, immune status, and treatment response based on survival analysis, immune cell infiltration, and drug sensitivity features.

## Materials and methods

### Localization and characterization of genes in the vicinity of 91 HERV-K sequences

We downloaded 91 HERV-K sequences (GenBank ID JN675007-JN675097) from the Gene Expression Omnibus (GEO-NCBI) database and BioMart from Ensembl (http://asia.ensembl.org/index.html) to search for genes located within a 60 kb range upstream and downstream of HERV-K.

### Collection and processing of GEO data

Nine RNA-seq data were obtained from GEO and NCBI SRA, which were original data from nine laboratories respectively, including GSE52194 (non-TNBC, HER2 + , TNBC), GSE45419 (ER + , HER2 + , TNBC), GSE103001 (ER +), GSE58135 (ER + , TNBC), GSE133998 (HER2 +), GSE183947 (MBC), GSE171957 (MDA-MB-231 and HCC1937 cells), GSE96860 (AU565, HCC1954, MDA-MB-231, MB436, MB468, HCC1937 cells), and GSE111842 (MCF7, ZR751, MB361, UACC812, SKBR3 cells).

The study included 199 clinically invasive breast cancer tissue samples, 11 normal breast controls from healthy donors, 44 BRCA cell samples, and eight normal control cell samples. Detailed information on each dataset is presented in Additional file 1: Table S1. A combined index was constructed using 91 HERV-K and human reference genome sequences (http://ftp.ensembl.org/pub/release-106/fasta/homo_sapiens/cdna/Homo_sapiens.GRCh38.cdna.all.fa.gz). Subsequently, the Salmon software was used to map the raw sequencing data to this index, followed by integration using the tximport R package. Differential analysis of HERV-K and genes in breast cancer samples was then conducted using the DESeq2 R package, with the screening criteria set at p < 0.05, and |log2(fc)|> 1.5.

### Select and visual candidate genes related to breast cancer prognosis from the neighborhood genes of HERV-K

The criteria for gene selection were as follows: (i) candidate genes were neighborhood genes of HERV-K; (ii) both candidate genes and HERV-K exhibited differential expression between breast cancer and normal control samples; and (iii) the expression of candidate genes was significantly correlated with that of HERV-K. Based on the aberrant activation expression of HERV-K among different samples, each HERV-K and its corresponding neighboring genes were considered as one group. The corresponding neighborhood genes were screened from DEGs, and the Pearson correlation coefficient was calculated. A correlation heat map was used to display the analysis results visually. There was a significant correlation between the expression of HERV-K and its neighboring genes, and the corresponding correlation coefficient appeared in the corresponding heat map region (p < 0.05). In this step, the R language "ggcorrplot" software package was used to complete the analysis. The selected genes from different datasets were visualized using a clustering heatmap and Venn diagram.

Crossover genes that appear in at least two breast cancer subtypes have been identified as candidate genes related to breast cancer prognosis and treatment. The Gene Ontology (GO) enrichment analysis of candidate genes was performed using the "clusterProfiler" R software package [26].

### Survival analysis and mutation burden analysis

Breast cancer samples were obtained in the FPKM format of RNA-seq data, MAF data, and relevant clinical data from the TCGA database, including survival status, staging, gender, and age. In addition, gene expression profiles of the control group (non-tumor samples) were acquired using the Genome Tissue Expression (GTEx) project (https://www.gtexportal.org). Validation of candidate genes for high-risk prognosis through an integrated analysis of GEO and TCGA data [27]. A multivariate Cox regression model was used to estimate overall survival (OS), and Kaplan–Meier curves were used to analyze the relationship between OS and the candidate genes. Further validation of the effectiveness and accuracy of the candidate genes in predicting 5-year prognosis was performed using ROC curves [28]. Tumor mutation burden (TMB) analysis is carried out using the "maftools" R package [29].

### Immune infiltration analysis

The TIMER online platform (https://cistrome.shinyapps.io/timer/) enables users to explore the correlation between immune cell infiltration and various factors, including somatic copy number alterations, somatic mutations, gene expression, and clinical outcomes. Users can use this platform to analyze the abundance of immune cell infiltration in different types of cancer and its association with different genomic and clinical features [30]. The abundance of tumor-infiltrating IMmune cells (TIICs) can be inferred from the gene expression profile through the TIMER (Tumor IMmune Estimation Resource), and 6 TIICs subpopulations (CD4 + T cells, CD8 + T cells, macrophages, B cells,) can be provided During the development and progression of tumors, malignant cells and tumor-infiltrating immune cells (TIICs) interact through a variety of gene products and pathways that are critical to understanding the tumor's immune environment and immune response TIMER can help researchers study and understand the complexity of tumor immunology and provide important information for clinical treatment and prognostic evaluation. The GSCA database (http://bioinfo.life.hust.edu.cn/GSCA) was used for drug sensitivity analysis [31].

### RT-PCR and correlation analysis

Total RNA was extracted from normal breast epithelial cell MCF-10A,and breast cancer cell MCF-7, AU565, MB468, and MB231 using TRIzol (ThermoFisher). The RNA concentration was diluted to 200 ng/μL. cDNA was synthesized using the PrimeScript RT reagent Kit (Takara, RR037A).The RT-PCR reaction was performed using SYBR Premix Ex Taq (Takara, RR047A) and conducted on the StepOnePlus real-time PCR system (Applied Biosystems, 4,376,600). ACTIN gene was used as the internal control for gene expression analysis. The relative expression levels were calculated using the 2^−ΔΔCt^ method. Each experiment was performed in triplicate and repeated three times. The primer sequences used in this study are listed in Additional file 4: Table S4. Further, correlation analysis was performed to calculate the Pearson correlation coefficient for differentially expressed genes between normal breast epithelial cells and breast cancer cells.

## Results

### Distribution of HERV-K and neighboring genes in the human genome

**Fig. 1:**
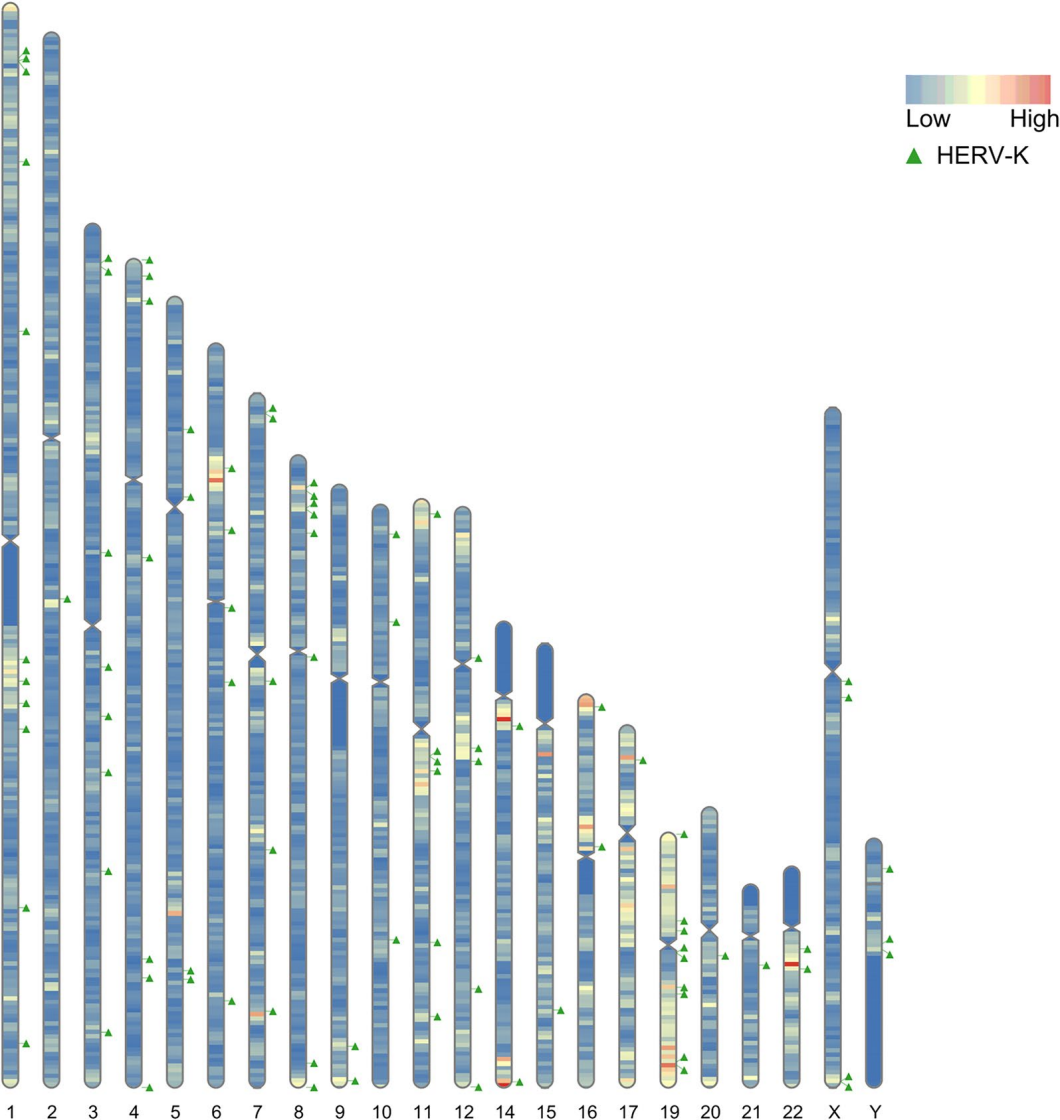
Distribution of 91 HERV-K on human chromosomes

Within the 60 kp upstream and downstream ranges of 91 human-specific HERV-Ks, there were 370 genes, including some pseudogenes. The distribution of host genes was relatively dense within the 0–35 kp sequence range around HERV-K. Additionally, over one-third of HERV-K reninsertions occur in certain host genes. The distribution of the 91 HERV-Ks in human chromosomes (Fig. 1) and the coordinates of the HERV-K loci and their corresponding neighboring host genes are shown in Additional file 2: Table S2.

### Differential expression analysis of DEGs and correlation analysis of HERV-K and neighboring gene expression between sample groups

Salmon software was used to map the raw sequencing data downloaded from the Gene Expression Omnibus and NCBI SRA to the 91 HERV-K genomes. Almost all HERV-Ks exhibited abnormal expression in breast cancer, with different expression patterns observed in each dataset. Based on the results of the correlation analyses between each abnormally expressed HERV-K and its neighboring genes, more than 10% of the HERV-K aberrantly activated in breast cancer correlated with the expression of neighboring genes. The relevant data included GSE52194, GSE45419, GSE103001, GSE171957, GSE58135, and GSE96860, and Heat maps were generated using R software (version 4.2.2) (Additional file 5: Figure S1).

**Fig. 2:**
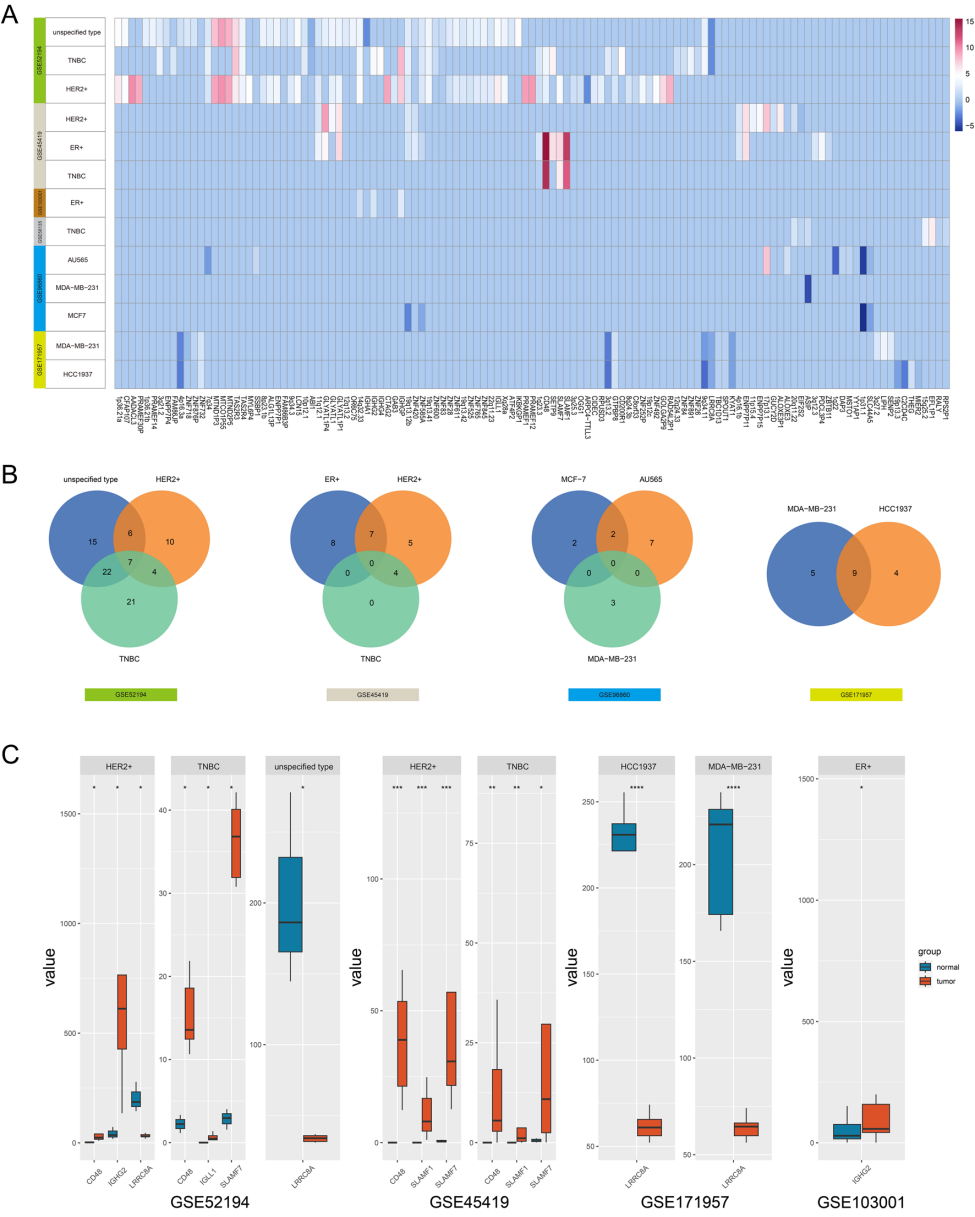
Distribution of abnormally activated HERV-K and its neighborhood candidate genes in different GSE datas. **A** Statistical results of HERV-K and its neighborhood candidate genes in GSE data, listed as HERV-K loci and neighborhood genes, and compared between breast cancer and normal control samples; The term 'unspecific type' refers to undifferentiated breast cancer samples. **B** Intersection of candidate genes and HERV-K between different samples in the GSE data. **C** Differential expression of 6 key genes in different subtypes of breast cancer

In the six GSE datasets, 78 genes showed a positive correlation with HERV-K expression, whereas 10 genes exhibited a negative correlation (Fig. 2A), among which 34 genes were identified in more than two subtypes of breast cancer samples (Fig. 2B). Among the six GSE datasets, 15 candidate HERV-K and 24 candidate genes were screened in GSE52194,4 candidate HERV-K, seven candidate genes in GSE45419, one candidate HERV-K, and one candidate gene in GSE96860, three candidate HERV-K, and six candidate genes in GSE171957. Ranked by |logFC|, LRRC8A was significantly underexpressed in ER + , HER2 + , and TNBC. IGHG2 expression is significantly higher in ER + and HER2 + breast cancer. CD48, SLAMF7, and SLAMF1 levels are significantly higher in HER2 + , and TNBC IGLL1 expression was significantly high expressed in ER + and HER2 + breast cancer cells (Fig. 2C).

### High expression of neighboring candidate genes of HERV-K is associated with poorprognosis in breast cancer

**Fig. 3:**
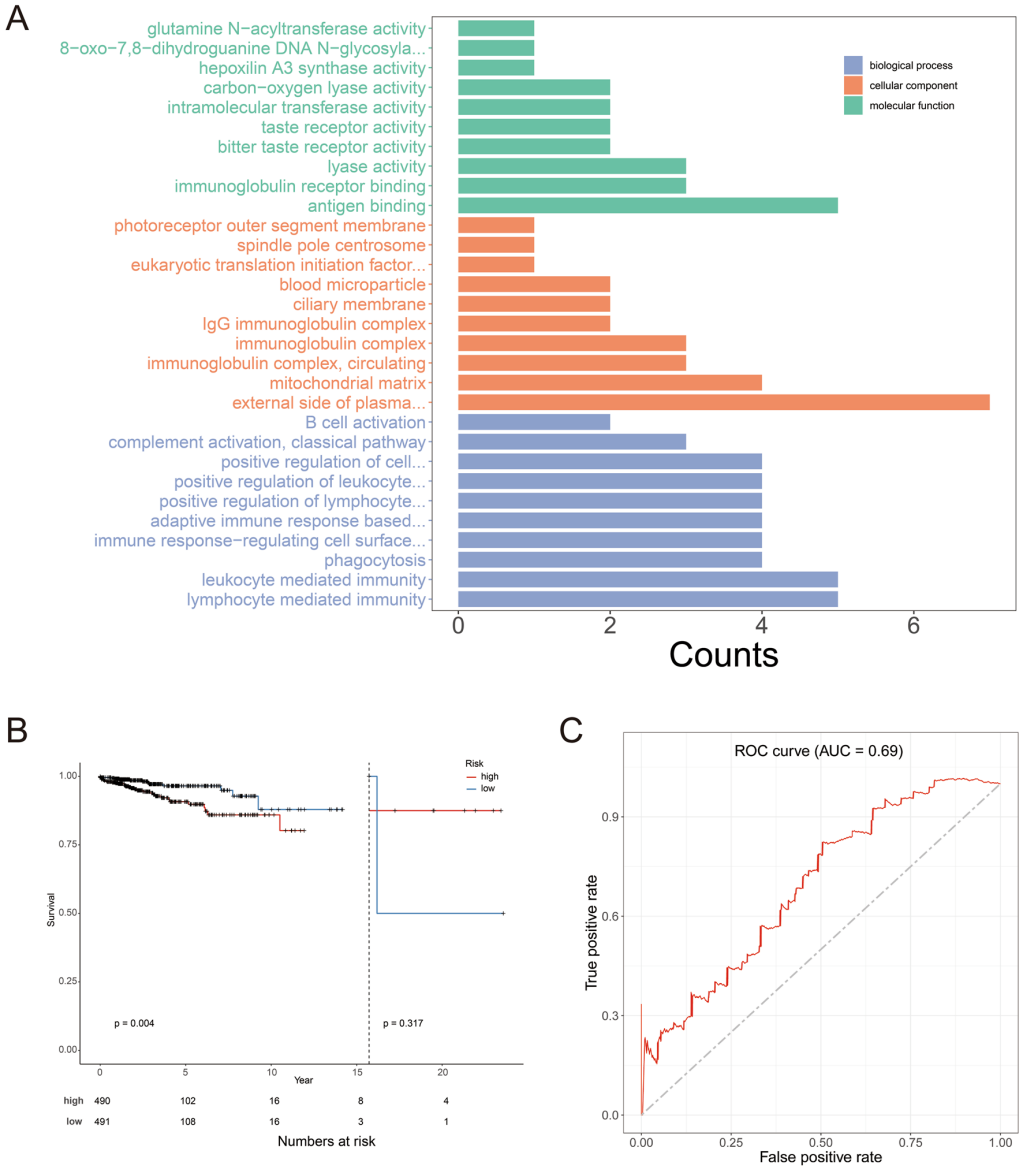
Functional enrichment analysis and prognostic analysis of candidate genes. **A** GO analysis of co-expression genes. **B** Survival curves were generated for patients with high expression (in red) and low expression (in blue) of the candidate genes. **C** The diagnostic value of candidate gene in BRCA patients. AUC: area under curve

Functional enrichment analysis revealed that the 34 candidate genes were primarily associated with lymphocyte-mediated immunity, leukocyte-mediated immunity, phagocytosis, immune response regulatory cell surface receptor signaling pathway, B cell activation, pre-B cell differentiation, and other pathways related to immune function (Fig. 3A). Further screening revealed six key genes involved in immune regulation, namely, IGHG2, IGHG4, IGLL1, SLAMF7, SLAMF1, CD48, and IGHA1, LRRC8A. These genes were strongly correlated with the expression of their corresponding neighboring HERV-K genes. The teams included SLAMF7, SLAMF1, and CD48 with HERV-K_1q23.3, IGHG2 with HERV-K_14q32.33, IGLL1 with HERV-K_22q11.23, IGHA1 with HERV-K_14q32.33, and LRRC8A with HERV-K_9q34.11. The risk score for each breast cancer patient was calculated using multivariable Cox regression analysis. Based on the risk score, the patients were divided into two groups: a high-risk group consisting of 490 patients and a low-risk group consisting of 491 patients. Kaplan–Meier analysis revealed that the low-risk group had a significantly higher survival rate than the high-risk group (p = 0.004) (Fig. 3B). The Area Under the Curve (AUC) of the ROC curve is 0.69, indicating that the prognostic risk model exhibits moderate effectiveness in predicting survival outcomes (Fig. 3C).

### Correlation between the expression of immune-related genes and immune cell infiltration in breast cancer microenvironment

**Fig. 4:**
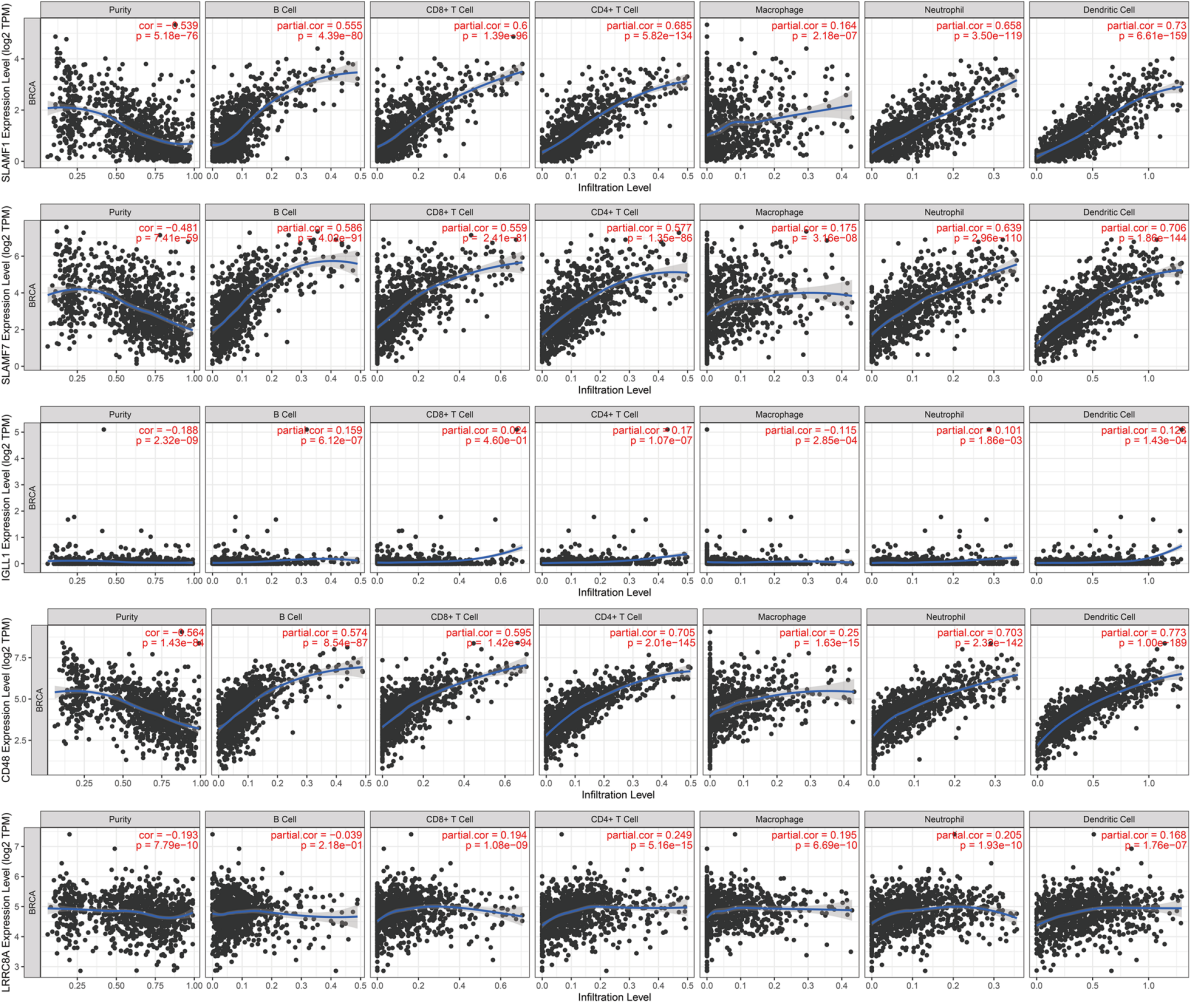
The association between IGLL1 SLAMF7 SLAMF1 CD48 LRRC8A expression and immune cell infiltrations

Tumor immune infiltration into the tumor microenvironment is a critical factor that influences the effectiveness of cancer treatment and patient prognosis [32]. We evaluated the correlation between the expression of IGLL1, SLAMF7, SLAMF1, CD48, and LRRC8A and the immune infiltration profile in breast cancer using data downloaded from TCGA. The results showed that IGLL1, SLAMF7, SLAMF1, CD48, and LRRC8A were closely associated with immune infiltration in breast cancer (*p* < 0.05, Fig. 4). Among the 24 types of infiltrating immune cells, B cells, CD8 + T cells, CD4 + T cells, neutrophils, macrophages, and dendritic cells were strongly positively correlated with the expression of LRRC8A, SLAMF7, SLAMF1, and CD48 genes (*p* < 0.05). The IGLL1 gene shows positive correlation with these immune cells, but its expression level is low and the correlation is weak.

### Mutation analysis and drug sensitivity analysis

**Fig. 5:**
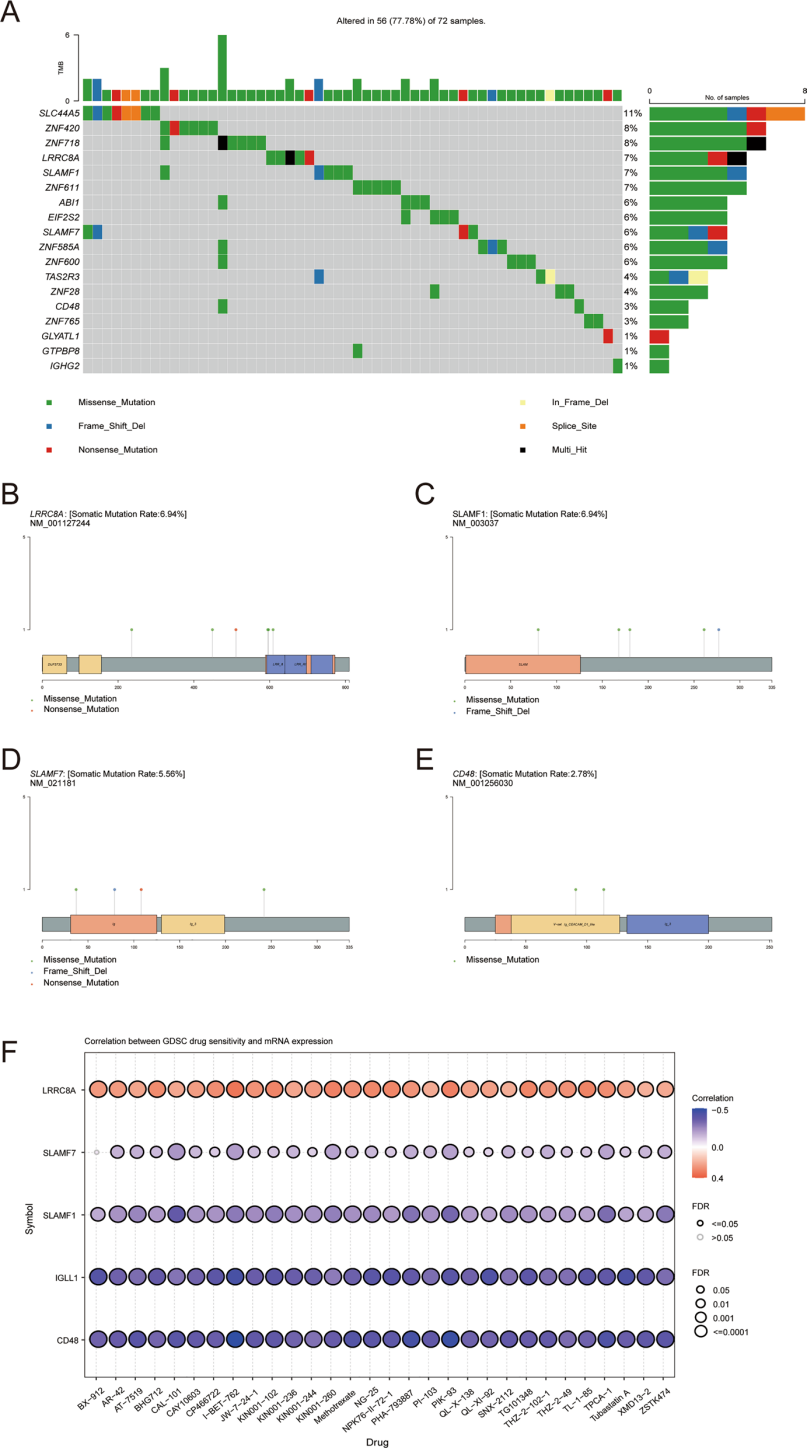
Immune-related gene mutation analysis and drug sensitivity analysis. **A** Related gene mutations. **B** LRRC8A gene mutation status. **C** SLAMF1 gene mutation status. **D** SLAMF7 gene mutation status. **E** CD48 gene mutation status. **F** GDSC database was used to analyze the correlation between drug sensitivity and immune-related gene expression

Further mutation analysis on the 34 genes was performed using the "maftools" R package. A waterfall plot was generated to show the 18 genes with the highest mutation rates, with different colors indicating different mutation types (Fig. 5A). Immune-related genes (SLAMF1, SLAMF7, and LRRC8A) had relatively high mutation frequencies.

In LRRC8A, six mutation sites were detected within amino acids 0–800 of the LRRC8A protein. These mutations consisted of five missense and one nonsense mutation (Fig. 5B). In SLAMF1, five mutation sites were identified within amino acids 0–335 of the SLAMF1 protein. Among them, there were four missense mutations and one frameshift deletion (Fig. 5C). In SLAMF7, four mutation sites were identified within amino acids 0–335 of the SLAMF7 protein. These mutations include two missense mutations, one frameshift deletion, and one nonsense mutation (Fig. 5D). In CD48, two missense mutation sites were found within amino acids 0–250 of the CD48 protein (Fig. 5E). To gain further insights into the clinical relevance of the upregulated expression levels of immune-related genes, namely IGLL1, SLAMF7, SLAMF1, and CD48, we utilized the GSCA database to investigate the potential associations between their expression levels and drug sensitivity (Fig. 5F). Lower expression of the LRRC8A gene is associated with reduced sensitivity of breast cancer patients to chemotherapy drugs such as BX-912, AR-42, AT-7519, BHG712, CAL-101, and CAY10603. Conversely, higher expression levels of IGLL1, SLAMF7, SLAMF1, and CD48 have been linked to decreased sensitivity to chemotherapeutic drugs.

### The activated expression of HERV-K influences the expression of neighboring genes

**Fig. 6:**
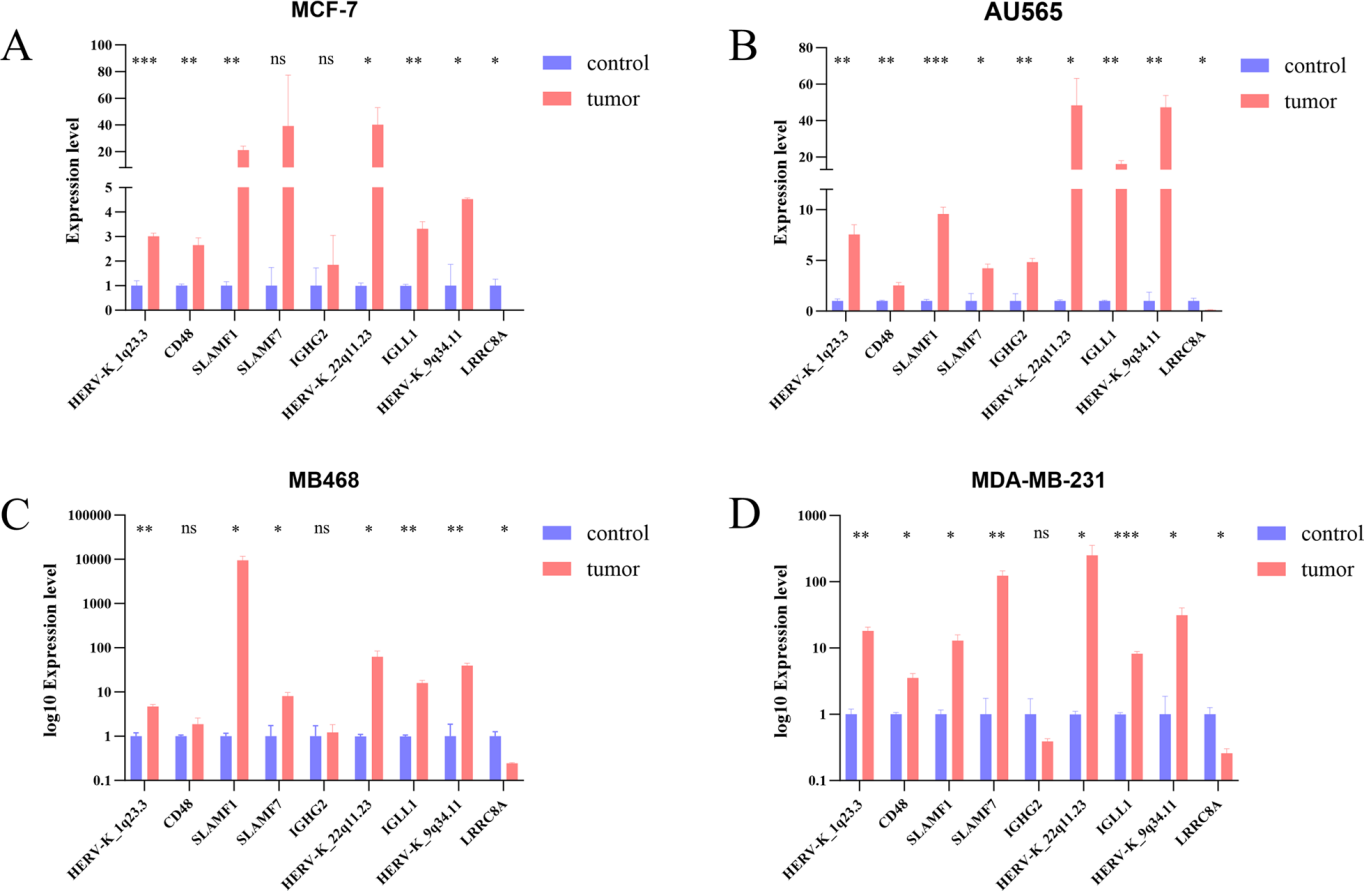
RT-PCR Analysis of Gene Expression in Various Cell Types: The red bars represent tumor cells, and the blue bars represent normal breast epithelial cells (MCF-10A). The X-axis represents different HERV-K and neighboring genes, while the Y-axis represents gene expression levels. **A** Gene expression levels in MCF-10A and MCF-7. **B** Gene expression levels in MCF-10A and AU565. **C** Gene expression levels in MCF-10A and MB468. **D** Gene expression levels in MCF-10A and MB231

Comparing the gene expression between normal breast epithelial cells and four types of breast cancer cells, it was found that all four different types of breast cancer cells exhibited differential expression of theHERV-K_1q23.3, SLAMF1, HERV-K_22q11.23, IGLL1, HERV-K_9q34.11 and LRRC8A. In three different types of breast cancer cells, differential expression was also observed in genes including HERV-K_1q23.3, CD48, SLAMF1, SLAMF7, HERV-K_22q11.23, IGLL1, HERV-K_9q34.11, and LRRC8A (Fig. 6). Further correlation analysis was performed for differentially expressed HERV-K and their adjacent genes. The results demonstrated consistency with the aforementioned bioinformatics analysis, showing that differentially expressed HERV-K and their adjacent genes include HERV-K_1q23.3 with CD48, SLAMF1, and SLAMF7; HERV-K_22q11.23 with IGLL1; and HERV-K_9q34.11 with LRRC8A (Additional file 6: Figure S2).

## Discussion

In the field of epigenetics research, human endogenous retrovirus (ERV) sequences are considered to play a crucial role in regulating gene expression and preserving genome stability. HERV-K elements can influence the expression levels of neighboring genes through diverse regulatory mechanisms, such as DNA methylation, histone modifications, and non-coding RNA interactions. These regulatory processes play critical roles in tumor occurrence and development. Understanding the impact of HERV-K elements on nearby gene expression is crucial for unraveling their potential implications in cancer biology and disease progression [12, 33, 34]. Here, we report for the first time the regulatory potential of 91 HERV-K proviruses on neighboring genes following abnormal activation in breast cancer. HERV-K may mediate the transcription of neighboring genes through proximal regulatory functions. HML-2 is considered the most recently integrated family of HERV-K elements, is the most biologically active subgroup within the HERV-K family, and possesses distinctive features in the human genome [14, 35], which provides a good system for exploring the regulatory function of HERV-K in neighboring genomic regions.

Constantly emerging evidence indicates that the genomic coordinates of HERV-K and sequence information of different insertion sites are being explored in the human genome. HERV-K sequences exist in two main forms in the human genome: one with a complete or nearly complete proviral sequence and the other composed only of LTR sequences [36]. Some insertion loci are referred to as proviral insertion sites because they contain one or more coding sequences for viral proteins. Indeed, genes located in the 0–35 kp region adjacent to HERV-K sequences in the human genome are known to be relatively densely distributed. One intriguing possibility is that the provirus, which is viral DNA integrated into the host genome, might undergo gene fusion with its neighboring host gene. This fusion event might have resulted in the creation of novel chimeric genes with potentially altered functions. Further research in this area could shed more light on the functional consequences of these potential gene fusions and their implications for human biology and health [37]. A total of 34 candidate genes were selected and screened to predict the prognosis of breast cancer effectively (p = 0.004). These genes were carefully chosen based on their potential association with HERV-K expression and involvement in immune-related functions. The goal was to identify genes that could serve as reliable prognostic indicators of breast cancer, allowing for a more accurate prediction of patient outcomes and disease progression.

Through rigorous analysis and validation, we aimed to determine the significance of 34 candidate genes in breast cancer prognosis and patient survival. CD48, SLAMF7, IGLL1, SLAMF1, IGHG2 and LRRC8A, deserve more investigations. These six genes were significantly enriched in immune function-related pathways. Among the 34 candidate genes, CD48 showed the highest expression (logFC = 15.52), followed by SLAMF1 (logFC = 13.35). All six key genes were identified as candidate genes in two or more samples. IGHG2 exhibited high expression in estrogen receptor-positive) and HER2 + (and human epidermal growth factor receptor 2-positive) breast cancer. On the other hand, CD48, SLAMF1, and SLAMF7 were highly expressed in HER2 + and triple-negative breast cancer (TNBC), and IGLL1 was highly expressed in ER + and TNBC tissues; These six key genes are closely associated with immune infiltration in breast cancer. In breast cancer, the high expression of SLAMF1, SLAMF7, CD48, and IGLL1 is positively correlated with the infiltration of B cells, CD8 + T cells, CD4 + T cells, macrophages, neutrophils, and dendritic cells. Conversely, the expression of LRRC8A is negatively correlated with the infiltration of the aforementioned immune cells. This suggests that the high expression of SLAMF1, SLAMF7, CD48, and IGLL1, as well as the low expression of LRRC8A, may induce or participate in activating the immune response within breast cancer tissue, regulating the activity and quantity of immune cells, thereby influencing the recognition and attack of tumor cells by immune cells. This infiltration of immune cells may be associated with the malignancy and prognosis of the tumor. IGLL1 showed a positive correlation with these immune cells, but its expression level was low, and the correlation was weak. However, the involvement of this gene in immune escape remains unclear. Further research is needed to investigate these results, and the results of the drug sensitivity analysis indicated that IGLL1, SLAMF7, SLAMF1, CD48, and LRRC8A might decrease sensitivity to specific chemotherapy drugs, further affecting the treatment efficacy for breast cancer. These variations in gene expression levels may influence the selection of treatment strategies and the prediction of therapeutic effectiveness. Further research is needed to elucidate the underlying mechanisms and potential clinical implications of this combination. Through in vitro experiments, we have confirmed that the activation of HERV-K in different subtypes of breast cancer cells (MCF-7, AU565, MDA-MB-231, and MB468) has a regulatory effect on neighboring genes. We observed certain differences in the expression of HERV-K_1q23.3, HERV-K_22q11.23, HERV-K_9q34.11, and HERV-K_14q32.33 and their adjacent genes in different breast cancer cell lines, which may be related to the heterogeneity of breast cancer cells [38, 39]. Furthermore, no differential expression was observed in HERV-K_14q32.33 across the four cell lines. However, a comprehensive analysis of gene expression results clearly indicates significant differential expression of HERV-K_1q23.3, SLAMF1, IGHG2, HERV-K_22q11.23, IGLL1, HERV-K_9q34.11, and LRRC8A in breast cancer cells, with a significant correlation between HERV-K and its corresponding neighboring genes. This finding is consistent with the trends identified in previous bioinformatics analyses.

The SLAM family (SLAMF) is a group of cell surface receptors that are involved in co-stimulation, cytokine production, and cytotoxicity and are crucial for regulating immune responses and facilitating communication between different immune cells [40–42]. The SLAM family (SLAMF) consists of nine cell surface receptors: CD150 (SLAM, SLAMF1), CD48 (SLAMF2, BLAST-1), CD229 (SLAMF3, Ly9), CD244 (SLAMF4, 2B4), CD84 (SLAMF5), CD352 (SLAMF6, Ly108, NTB-A), CD319 (SLAMF7, CRACC), CD353 (SLAMF8, BLAME), and CD84H (SLAMF9, SF2001). These receptors are involved in various aspects of immune function, such as co-stimulation, cytokine production, and cytotoxicity. Among the SLAM family members, CD48 (SLAMF2 and BLAST-1) plays a primary role in the adhesion and activation of immune cells. It is expressed on the surfaces of different immune cells and is crucial for mediating interactions between these cells. CD48 is involved in immune cell signaling and regulation, and its functions are essential for coordinating immune responses and promoting effective immune cell communication [43]. In the immune system, CD48 was the first discovered receptor for growth differentiation factor 15 (GDF15), and studies have shown that upregulated expression of GDF15 in HCC can regulate the suppressive function of natural Tregs (nTregs) through interaction with the CD48 receptor on T cells and transcriptional gene silencing mechanisms [44]. SLAMF1 is a co-stimulatory molecule involved in immune regulation that participates in host innate and adaptive responses [45]. In cancers like Hodgkin's lymphoma and chronic lymphocytic leukemia, IGLL1 also plays a crucial role in regulating the tumor microenvironment and determining the fate of malignant cells. Its involvement in these processes renders it a potential target for cancer treatment and further research [46]. SLAMF7 (also known as CD319, CRACC, and CS1) plays a central role in highly activated macrophage-related inflammatory diseases [36, 40]. SLAMF7 activation in inflammatory macrophages is a key pathway driving the pathology of acute and chronic inflammatory human diseases [47]. IGLL1, also known as immunoglobulin lambda-like polypeptide 1, is a member of the immunoglobulin gene superfamily that plays a vital role in B cell development [48]. IGHG2 (immunoglobulin heavy constant γ2) is a protein-coding gene that is associated with certain diseases such as immunoglobulin kappa light chain deficiency. Related pathways involve the production of C4 and C2 activators and the innate immune system [49]. Leucine-rich repeat protein A (LRRC8A), also known as SWELL1, is a core component of anionic channels (VRAC) [50]. LRRC8A is closely associated with the occurrence of multiple tumors [51–53].

An imbalance in the tumor immune microenvironment (TME) is one of the most significant characteristics of tumors [54]. The TME contains a variety of cell types, including tumor cells, stromal cells, and immune cells (T cells, B cells, and macrophages) [55]. The adaptive immune response mediated by immune cells plays a key role in tumor progression [56]. In various cancer types, the infiltration of immune cell populations has shown diverse prognostic outcomes, with immune cell types such as CD8 + T cells, B cells, CD4 + T cells, and neutrophil-macrophage dendritic cells playing crucial roles in the progression of specific tumors and influencing the response to immunotherapy [57–59]. In a small number of tumors, the infiltration of innate immune-related cells, such as natural killer (NK) cells, bone marrow-derived suppressor cells (MDSC), and DCs, is associated with prognosis, but the difference is great. Macrophage infiltration is a hallmark of solid cancers, and overall macrophage infiltration is associated with lower patient survival and treatment resistance [59]. The M1-type macrophages and M2-type macrophages are two distinct polarized subtypes of macrophages. M1-type macrophages are typically associated with antitumor immune responses and inflammation. Conversely, M2-type macrophages are associated with anti-inflammatory responses, immune regulation, and tumor growth[60]. The development of tumors is often associated with an imbalance between M1/M2-type macrophages[61, 62]. HERV-K may regulate immune responses, tumor progression, and treatment outcomes in breast cancer by potentially influencing macrophage activity and phenotype. Additionally, neutrophil mast cells and eosinophils have been associated with several tumor outcomes, with high neutrophil infiltration predicting poor prognosis and mast cells and eosinophils predicting good prognosis[63]. Therefore, we propose that the expression of immune-related genes in the breast cancer microenvironment is closely associated with immune cell infiltration. HERV-K_1q23.3, HERV-K_14q32.33, HERV-K_22q11.23, and HERV-K_9q34.11 may be markers of immune infiltration and poor prognosis in breast cancer.

Interestingly, the HERV-K sequence at locus 1q23.3, was completely integrated within the exon of CD48, with a 99.98% overlap with the CD48 gene sequence. The protovirus is 9232 bp in length, and with 5′LTR-gag-pol-env-3′LTR structure, we speculate that the change of CD48 expression is closely related to the insertion of HERVK_1q23.3 into the CD48 sequence. In addition, CD48, SLAMF7, IGLL1, SLAMF1, IGHG2, and LRRC8A showed different degrees of mutation. This may be another way in which HERV-K in the human genome shapes host genes [64].

Here, we found that in breast cancer samples, abnormally activated HERV-K viruses upregulated the expression of certain neighboring genes and downregulated the expression of certain genes. Of course, the ability to upregulate the expression of neighboring genes is more significant. For example, HERV-K_14q32.33 not only upregulates the expression of its upstream neighboring gene IGHG2 but also downregulates the expression of its downstream neighboring gene IGHAI. According to previous reports, HERV-K has a bidirectional promoter activity. We speculate that the potential antisense transcript at 14q32.33 may downregulate the expression of the IGHAI transcript [65]. These observations strongly support the hypothesis of diversity in HERV-regulated host gene pathways [12]. Further studies are needed to investigate how the original HERV-K viruses regulate their neighboring genes.

Our study extensively utilized publicly available raw data from nine laboratories to ensure high credibility and richness of the samples. These data substantially reflect the abnormal expression patterns of HERV-Ks in breast cancer, providing a substantial basis for further investigating the regulatory relationship between abnormal HERV-K expression and neighboring immune-related genes. However, it is essential to acknowledge the inherent limitations of bioinformatics analysis, despite our careful efforts and experimental validation. Moreover, whether our findings can be extrapolated to other types of tumors requires further validation and research.

## Conclusion

In summary, this study investigated for the first time the regulatory effects of 91 HERV-K proviruses on the neighboring genomic regions in breast cancer. The research revealed their relationship with clinical prognosis, immune cell infiltration, and their roles in the progression of breast cancer. Despite certain limitations in bioinformatics, our data analysis and experimental validation preliminarily identified three critical HERV-K proviruses and five proximal genes closely associated with immune cell infiltration and prognosis in breast cancer. These findings offer new targets to advance more precise and personalized immune therapies for breast cancer.

### Supplementary Information

**Additional file 1: Table S1.** The character of nine expression profiling datasets downloaded from SRA/NCBI.

**Additional file 2: Table S2.** Presence of host genes in the upstream and downstream 60kb range of HERV-Ks in the human genome.

**Additional file 3: Table S3.** Statistical results of HERV-Ks and their neighborhood candidate genes in GSE data.

**Additional file 4: Table S4.** Primer sequences.

**Additional file 5: Figure S1.** Correlation of HERV-K provirus expression with neighboring genomes in various GSE dataset samples.

**Additional file 6: Figure S2.** Correlation analysis of HERV-K with neighboring genes.

## Data Availability

The breast cancer tissue and cell RNA-seq mentioned in this paper were obtained from GEO and NCBI SRA public databases. For details of the original data, please refer to additional materials (Additional file 1: Table S1).

## References

[1] Cao W, Chen HD, Yu YW (2021). Changing profiles of cancer burden worldwide and in China: a secondary analysis of the global cancer statistics 2020. Chin Med J.

[2] Siegel RL, Miller KD, Fuchs HE (2022). Cancer statistics. Cancer J Clin.

[3] Sung H, Ferlay J, Siegel RL (2021). Global Cancer Statistics 2020: GLOBOCAN Estimates of Incidence and Mortality Worldwide for 36 Cancers in 185 Countries. Cancer J Clin.

[4] Tavakolian S, Goudarzi H, Faghihloo E (2019). Evaluating the expression level of HERV-K env, np9, rec and gag in breast tissue. Infectious Agents Cancer..

[5] Kaplan MH, Contreras-Galindo R, Jiagge E (2020). Is the HERV-K HML-2 Xq21.33, an endogenous retrovirus mutated by gene conversion of chromosome X in a subset of African populations, associated with human breast cancer?. Infectious Agents Cancer..

[6] Johanning GL, Malouf GG, Zheng X (2017). Expression of human endogenous retrovirus-K is strongly associated with the basal-like breast cancer phenotype. Sci Rep.

[7] Zhang X, Zhang R, Yu J (2020). New Understanding of the Relevant Role of LINE-1 Retrotransposition in Human Disease and Immune Modulation. Front Cell Develop Biol..

[8] Cao W, Kang R, Xiang Y (2020). Human endogenous retroviruses in clear cell renal cell carcinoma: biological functions and clinical values. OncoTargets Therapy..

[9] Maze EA, Agit B, Reeves S (2022). Human Endogenous Retrovirus Type K Promotes Proliferation and Confers Sensitivity to Antiretroviral Drugs in Merlin-Negative Schwannoma and Meningioma. Can Res.

[10] Doucet-O'Hare TT, DiSanza BL, DeMarino C (2021). SMARCB1 deletion in atypical teratoid rhabdoid tumors results in human endogenous retrovirus K (HML-2) expression. Sci Rep.

[11] Montesion M, Williams ZH, Subramanian RP (2018). Promoter expression of HERV-K (HML-2) provirus-derived sequences is related to LTR sequence variation and polymorphic transcription factor binding sites. Retrovirology.

[12] Zhang T, Zheng R, Li M (2022). Active endogenous retroviral elements in human pluripotent stem cells play a role in regulating host gene expression. Nucleic Acids Res.

[13] Buzdin A, Kovalskaya-Alexandrova E, Gogvadze E (2006). At least 50% of human-specific HERV-K (HML-2) long terminal repeats serve in vivo as active promoters for host nonrepetitive DNA transcription. J Virol.

[14] Garcia-Montojo M, Doucet-O'Hare T, Henderson L (2018). Human endogenous retrovirus-K (HML-2): a comprehensive review. Crit Rev Microbiol.

[15] Mueller T, Hantsch C, Volkmer I (2018). Differentiation-dependent regulation of human endogenous retrovirus K sequences and neighboring genes in germ cell tumor cells. Front Microbiol.

[16] Liu M, Jia L, Li H (2022). p53 Binding Sites in Long Terminal Repeat 5Hs (LTR5Hs) of Human Endogenous Retrovirus K Family (HML-2 Subgroup) play important roles in the regulation of LTR5Hs transcriptional activity. Microbiology Spectrum.

[17] Bannert N, Kurth R (2004). Retroelements and the human genome: new perspectives on an old relation. Proc Natl Acad Sci USA.

[18] Hohn O, Hanke K, Bannert N (2013). HERV-K(HML-2), the Best Preserved Family of HERVs: Endogenization, Expression, and Implications in Health and Disease. Front Oncol.

[19] Xue B, Sechi LA, Kelvin DJ (2020). Human Endogenous Retrovirus K (HML-2) in Health and Disease. Front Microbiol.

[20] Durnaoglu S, Kim HS, Ahnn J (2020). Human Endogenous Retrovirus K (HERV-K) can drive gene expression as a promoter in Caenorhabditis elegans. BMB Rep.

[21] Jern P, Coffin JM (2008). Effects of retroviruses on host genome function. Ann Rev Genetics..

[22] Xiang X, Tao Y, DiRusso J (2022). Human reproduction is regulated by retrotransposons derived from ancient Hominidae-specific viral infections. Nat Commun.

[23] Tomlins SA, Laxman B, Dhanasekaran SM (2007). Distinct classes of chromosomal rearrangements create oncogenic ETS gene fusions in prostate cancer. Nature.

[24] Xiao Y, Yu D (2021). Tumor microenvironment as a therapeutic target in cancer. Pharmacol Therapeut..

[25] Sahai E, Astsaturov I, Cukierman E (2020). A framework for advancing our understanding of cancer-associated fibroblasts. Nat Rev Cancer.

[26] Xiao Y, Zhang B, Cloyd JM (2022). Novel drug candidate prediction for intrahepatic cholangiocarcinoma via hub gene network analysis and connectivity mapping. Cancers.

[27] Zhang M, Ma J, Guo Q (2022). CD8(+) T cell-associated gene signature correlates with prognosis risk and immunotherapy response in patients with lung adenocarcinoma. Front Immunol.

[28] Wang W, Lu Z, Wang M (2022). The cuproptosis-related signature associated with the tumor environment and prognosis of patients with glioma. Front Immunol.

[29] Mayakonda A, Lin DC, Assenov Y (2018). Maftools: efficient and comprehensive analysis of somatic variants in cancer. Genome Res.

[30] Li T, Fan J, Wang B (2017). TIMER: A web server for comprehensive analysis of tumor-infiltrating immune cells. Can Res.

[31] Liu CJ, Hu FF, Xie GY (2023). GSCA: an integrated platform for gene set cancer analysis at genomic, pharmacogenomic and immunogenomic levels. Brief Bioinformat..

[32] Wang J, Liu W, Li JC (2021). Hepcidin Downregulation Correlates With Disease Aggressiveness And Immune Infiltration in Liver Cancers. Front Oncol.

[33] Chuong EB (2018). The placenta goes viral: Retroviruses control gene expression in pregnancy. PLoS Biol.

[34] Meyer TJ, Rosenkrantz JL, Carbone L (2017). Endogenous Retroviruses: With Us and against Us. Front Chem.

[35] Wildschutte JH, Williams ZH, Montesion M (2016). Discovery of unfixed endogenous retrovirus insertions in diverse human populations. Proc Natl Acad Sci USA.

[36] Xue B, Zeng T, Jia L (2020). Identification of the distribution of human endogenous retroviruses K (HML-2) by PCR-based target enrichment sequencing. Retrovirology.

[37] Imakawa K, Nakagawa S (2017). The Phylogeny of Placental Evolution Through Dynamic Integrations of Retrotransposons. Progr Mol Biol Transl Sci..

[38] Nolan E, Lindeman GJ, Visvader JE (2023). Deciphering breast cancer: from biology to the clinic. Cell.

[39] Pellacani D, Tan S, Lefort S (2019). Transcriptional regulation of normal human mammary cell heterogeneity and its perturbation in breast cancer. EMBO J.

[40] Calpe S, Wang N, Romero X (2008). The SLAM and SAP gene families control innate and adaptive immune responses. Advan Immunol..

[41] Cannons JL, Tangye SG, Schwartzberg PL (2011). SLAM family receptors and SAP adaptors in immunity. Ann Rev Immunol.

[42] Pahima H, Puzzovio PG, Levi-Schaffer F (2019). 2B4 and CD48: A powerful couple of the immune system. Clin Immunol.

[43] McArdel SL, Terhorst C, Sharpe AH (2016). Roles of CD48 in regulating immunity and tolerance. Clin Immunol.

[44] Wang Z, He L, Li W (2021). GDF15 induces immunosuppression via CD48 on regulatory T cells in hepatocellular carcinoma. J Immunother Cancer.

[45] Pellegrini JM, Sabbione F, Morelli MP (2021). Neutrophil autophagy during human active tuberculosis is modulated by SLAMF1. Autophagy.

[46] Gordiienko I, Shlapatska L, Kovalevska L (2019). SLAMF1/CD150 in hematologic malignancies: Silent marker or active player?. Clin Immunol.

[47] Simmons DP, Nguyen HN, Gomez-Rivas E (2022). SLAMF7 engagement superactivates macrophages in acute and chronic inflammation. Sci Immunol..

[48] Lee RD, Munro SA, Knutson TP (2021). Single-cell analysis identifies dynamic gene expression networks that govern B cell development and transformation. Nat Commun.

[49] Orwoll ES, Wiedrick J, Nielson CM (2020). Proteomic assessment of serum biomarkers of longevity in older men. Aging Cell.

[50] Qiu Z, Dubin AE, Mathur J (2014). SWELL1, a plasma membrane protein, is an essential component of volume-regulated anion channel. Cell.

[51] Chen Y, Zuo X, Wei Q (2023). Upregulation of LRRC8A by m(5)C modification-mediated mRNA stability suppresses apoptosis and facilitates tumorigenesis in cervical cancer. Int J Biol Sci.

[52] Kurashima K, Shiozaki A, Kudou M (2021). LRRC8A influences the growth of gastric cancer cells via the p53 signaling pathway. Gastric Cancer.

[53] Xu R, Hu Y, Xie Q (2022). LRRC8A Is a Promising Prognostic Biomarker and Therapeutic Target for Pancreatic Adenocarcinoma. Cancers.

[54] Bejarano L, Jordāo MJC, Joyce JA (2021). Therapeutic Targeting of the Tumor Microenvironment. Cancer Discov.

[55] Kwon Y, Kim M, Kim Y (2020). Exosomal MicroRNAs as Mediators of Cellular Interactions Between Cancer Cells and Macrophages. Front Immunol.

[56] Dey P, Kimmelman AC, DePinho RA (2021). Metabolic codependencies in the tumor microenvironment. Cancer Discov.

[57] Zhang Y, Zhang Z (2020). The history and advances in cancer immunotherapy: understanding the characteristics of tumor-infiltrating immune cells and their therapeutic implications. Cell Mol Immunol.

[58] Hiss S, Eckstein M, Segschneider P (2021). Tumour-Infiltrating Lymphocytes (TILs) and PD-L1 Expression Correlate with Lymph Node Metastasis, High-Grade Transformation and Shorter Metastasis-Free Survival in Patients with Acinic Cell Carcinoma (AciCC) of the Salivary Glands. Cancers.

[59] Nalio Ramos R, Missolo-Koussou Y, Gerber-Ferder Y (2022). Tissue-resident FOLR2(+) macrophages associate with CD8(+) T cell infiltration in human breast cancer. Cell.

[60] Eum HH, Kwon M, Ryu D (2020). Tumor-promoting macrophages prevail in malignant ascites of advanced gastric cancer. Exp Mol Med.

[61] Song H, Yang Y, Sun Y (2022). Circular RNA Cdyl promotes abdominal aortic aneurysm formation by inducing M1 macrophage polarization and M1-type inflammation. Mol Ther.

[62] Kashfi K, Kannikal J, Nath N (2021). Macrophage Reprogramming and Cancer Therapeutics: Role of iNOS-Derived NO. Cells.

[63] Zuo S, Wei M, Wang S (2020). Pan-Cancer Analysis of Immune Cell Infiltration Identifies a Prognostic Immune-Cell Characteristic Score (ICCS) in Lung Adenocarcinoma. Front Immunol.

[64] Stocking C, Kozak CA (2008). Murine endogenous retroviruses. Cellular and molecular life sciences : CMLS.

[65] Babaian A, Mager DL (2016). Endogenous retroviral promoter exaptation in human cancer. Mob DNA.

