# Supplementary material for: HERVK-mediated regulation of neighboring genes: implications for breast cancer prognosis
Source: Retrovirology. 2024 Feb 22;21:4. doi: 10.1186/s12977-024-00636-z (PMC10885364; doi:10.1186/s12977-024-00636-z)
Supplement: Supplementary file 1 — Additional file 1: Table S1. The character of nine expression profiling datasets downloaded from SRA/NCBI. [file 12977_2024_636_MOESM1_ESM.docx]

**Table S1 the character of nine expression profiling datasets downloaded from SRA/NCBI**

| **GenBankID**  (Cancer sample) | **Country** | **Platform** | **cDNA Library** | **Controls Samples** | | **Case Samples** | | **Filter condition** | **Reference** |
| --- | --- | --- | --- | --- | --- | --- | --- | --- | --- |
|  |  |  |  | **Number** | **Source** | **Number** | **Source** |  |  |
| GSE45419  (Primary Tumors) | USA | Illumina Genome Analyzer IIx | paired-end | 8 benign breast lesions | tissue | 8 ER+ BCa  8 HER2+ BCa  8 TNBC BCa | tissue | PE-Truseq2 | (1) |
| GSE111842  (Primary Tumors) | USA | Illumina HiSeq 2500 | paired-end | 6 healthy individuals | peripheral blood | 16 circulating tumor cells | blood | PE-Truseq3 | (2) |
|  |  |  |  |  |  | 12 BCa | tissue |  |  |
| GSE96860  (Cell Line) | USA | Illumina HiSeq 2000 | Singled-end | 4 76NF2V  4 MCF10A | cells | 4 MCF7  4 ZR751  4 MB361  4 UACC812  4 SKBR3  4 AU565  4 HCC1954  4 MB231  4 MB436  4 MB468  4 HCC1937 | cells | SE-Truseq3 | (3) |
| GSE171957  (Cell Line) | USA | Illumina NextSeq 500 | Singled-end | 3 MCF10A | cells | 3 HCC1937 | cells | SE-Truseq3 | (4) |
|  |  |  |  |  |  | 3 MDA-MB-231 | cells |  |  |
| GSE58135  (Cell Lines and Primary Tumors) | USA | Illumina HiSeq 2000 | paired-end | 30 adjacent to ER+ BCa  5 Reduction Mammoplasty  21 Reduction Mammoplasty | tissue | 42 ER+  42 TNBC | tissue | PE-Truseq3 | (5) |
|  |  |  |  |  |  | 28 BCa Cell Line | cells |  |  |
| GSE52194  (Primary Tumor) | USA | Illumina HiSeq 2000 | paired-end | 3 normal human breast organoids | tissue | 6 TNBC BCa  5 HER2+ BCa  6 non-TNBC BCa | tissue | Truseq3 | (6) |
| GSE183947  (Primary and Metastasis) | China | Illumina HiSeq 2000 | paired-end | 30 adjacent to BCa | tissue | 15 BCa(primary) | tissue | Truseq3 | (7) |
|  |  |  |  |  |  | 15 BCa(metastasis) | tissue |  |  |
| GSE133998  (Primary Tumor) | China | HiSeq X Ten | paired-end | 6 adjacent to BCa | tissue | 6 BCa | tissue | Truseq3 | (8) |
| GSE103001  (Primary Tumor) | Belgium | Illumina HiSeq 2000 | paired-end | 22 adjacent to BCa | tissue | 22 ER+ BCa | tissue | Truseq3 | (9) |

1. Kalari KR, Necela BM, Tang X, Thompson KJ et al. An integrated model of the transcriptome of HER2-positive breast cancer. PLoS One. 2013, 8(11):e79298.

2. Lang JE, Ring A, Porras T, Kaur P et al. RNA-Seq of Circulating Tumor Cells in Stage II-III Breast Cancer. Ann Surg Oncol 2018 Aug;25(8):2261-2270.

3. Franco HL, Nagari A, Malladi VS, Li W et al. Enhancer transcription reveals subtype-specific gene expression programs controlling breast cancer pathogenesis. Genome Res 2018 Feb;28(2):159-170.

4. Chappell K, Manna K, Washam CL, Graw S et al. Multi-omics data integration reveals correlated regulatory features of triple negative breast cancer. Mol Omics 2021 Oct 11;17(5):677-691.

5. Varley KE, Gertz J, Roberts BS, Davis NS et al. Recurrent read-through fusion transcripts in breast cancer. Breast Cancer Res Treat 2014 Jul;146(2):287-97.

6. Eswaran J, Cyanam D, Mudvari P, Reddy SD et al. Transcriptomic landscape of breast cancers through mRNA sequencing. Sci Rep 2012;2:264.

7. Yan Zhang,Gui-Hui Tong,Xu-Xuan Wei et al. Identification of Five Cytotoxicity-Related Genes Involved in the Progression of Triple-Negative Breast Cancer. Front Genet 2022 Jan 3;12:723477.

8. Xu X, Zhang J, Tian Y, Gao Y et al. CircRNA inhibits DNA damage repair by interacting with host gene. Mol Cancer 2020 Aug 24;19(1):128.

9. Wenric S, ElGuendi S, Caberg JH, Bezzaou W et al. Transcriptome-wide analysis of natural antisense transcripts shows their potential role in breast cancer. Sci Rep 2017 Dec 12;7(1):17452.
