# Supplementary material for: HERVK-mediated regulation of neighboring genes: implications for breast cancer prognosis
Source: Retrovirology. 2024 Feb 22;21:4. doi: 10.1186/s12977-024-00636-z (PMC10885364; doi:10.1186/s12977-024-00636-z)
Supplement: Supplementary file 2 — Additional file 2: Table S2. Presence of host genes in the upstream and downstream 60kb range of HERV-Ks in the human genome. [file 12977_2024_636_MOESM2_ESM.docx]

| **Table S2 Presence of host genes in the upstream and downstream 60kb range of HERV-Ks in the human genome**  人类基因组中HERV-Ks上下游60kb范围内宿主基因的存在情况 | | | | | |
| --- | --- | --- | --- | --- | --- |
| **GenBank ID** | **Chromosomal region** | **Coordinate** | **0-35kb genome** | **35-60kb**  **genome** | **The internal genome of HERV-Ks** |
| JN675007.1 | 1p31.1 | chr1:75377086-75383458 |  |  | SLC44A5 |
| JN675008.1 | 1p34.3 | chr1:36488984-36491127 | MRPS15  CSF3R | [OSCP1](http://asia.ensembl.org/homo_sapiens/Gene/Summary?db=core;g=ENSG00000116885) |  |
| JN675009.1 | 1p36.21a | chr1:12780115-12785935 | [CFAP107](http://asia.ensembl.org/homo_sapiens/Gene/Summary?db=core;g=ENSG00000157330)  PRAMEF12  PRAMEF1 | [AADACL3](http://asia.ensembl.org/homo_sapiens/Gene/Summary?db=core;g=ENSG00000188984)  [LINC01784](http://asia.ensembl.org/homo_sapiens/Gene/Summary?db=core;g=ENSG00000228338)  [PRAMEF11](http://asia.ensembl.org/homo_sapiens/Gene/Summary?db=core;g=ENSG00000239810)  [PRAMEF30P](http://asia.ensembl.org/homo_sapiens/Gene/Summary?db=core;g=ENSG00000231103) |  |
| JN675010.1 | 1p36.21b | chr1:13352736-13362257 | [PRAMEF15](http://asia.ensembl.org/homo_sapiens/Gene/Summary?db=core;g=ENSG00000204501)  [PRAMEF14](http://asia.ensembl.org/homo_sapiens/Gene/Summary?db=core;g=ENSG00000204481)  PRAMEF19  PRAMEF17 | [PRAMEF33](http://asia.ensembl.org/homo_sapiens/Gene/Summary?db=core;g=ENSG00000237700)  [PRAMEF20](http://asia.ensembl.org/homo_sapiens/Gene/Summary?db=core;g=ENSG00000204478) |  |
| JN675011.1 | 1p36.21c | chr1:13352736-13361837 | PRAMEF15  PRAMEF14  PRAMEF19  PRAMEF17 | [PRAMEF33](http://asia.ensembl.org/homo_sapiens/Gene/Summary?db=core;g=ENSG00000237700)  [PRAMEF20](http://asia.ensembl.org/homo_sapiens/Gene/Summary?db=core;g=ENSG00000204478) |  |
| JN675012.1 | 1q21.3 | chr1:150632885-150634662 | ENSA  U4  GOLPH3L | [MCL1](http://asia.ensembl.org/homo_sapiens/Gene/Summary?db=core;g=ENSG00000143384) |  |
| JN675013.1 | 1q23.3 | chr1:160690785-160700016 | SETP9 | [SLAMF1](http://asia.ensembl.org/homo_sapiens/Gene/Summary?db=core;g=ENSG00000117090)  [SLAMF7](http://asia.ensembl.org/homo_sapiens/Gene/Summary?db=core;g=ENSG00000026751) | CD48 |
| JN675014.1 | 1q22 | chr1:155626666-155635845 | DAP3P1  MSTO1  YY1AP1 | [DAP3](http://asia.ensembl.org/homo_sapiens/Gene/Summary?db=core;g=ENSG00000132676) |  |
| JN675015.1 | 1q24.1 | chr1:166605366-166611021 | FMO8P | [FMO10P](http://asia.ensembl.org/homo_sapiens/Gene/Summary?db=core;g=ENSG00000234984) | FMO9P |
| JN675017.1 | 1q43 | chr1:238762295-238764473 | MIPEPP2 |  |  |
| JN675018.1 | 2q21.1 | chr2:129961965-129965044 | PLAC9P1  LINC01856  RPL22P7  RAB6C-AS1  RAB6C | [ARHGAP42P2](http://asia.ensembl.org/homo_sapiens/Gene/Summary?db=core;g=ENSG00000231147) |  |
| JN675019.1 | 3p12.3 | chr3:75551314-75559999 | LINC02018  SNRPCP10  RPS3AP15 | [OR7E121P](http://asia.ensembl.org/homo_sapiens/Gene/Summary?db=core;g=ENSG00000244222)  [UNC93B3](http://asia.ensembl.org/homo_sapiens/Gene/Summary?db=core;g=ENSG00000172971) | NEPRO-AS1 |
| JN675020.1 | 3p25.3 | chr3:9847662-9854552 | ARPC4-TTLL3  RPUSD3  CIDEC | [OGG1](http://asia.ensembl.org/homo_sapiens/Gene/Summary?db=core;g=ENSG00000114026)  [TADA3](http://asia.ensembl.org/homo_sapiens/Gene/Summary?db=core;g=ENSG00000171148)  [JAGN1](http://asia.ensembl.org/homo_sapiens/Gene/Summary?db=core;g=ENSG00000171135)  [IL17RE](http://asia.ensembl.org/homo_sapiens/Gene/Summary?db=core;g=ENSG00000163701) | TTLL3 |
| JN675021.1 | 3q12.3 | chr3:101691893-101701015 | ZBTB11  RNY1P12 | [Y_RNA](http://asia.ensembl.org/homo_sapiens/Gene/Summary?db=core;g=ENSG00000201511)  [ZBTB11-AS1](http://asia.ensembl.org/homo_sapiens/Gene/Summary?db=core;g=ENSG00000256628)  [RPL24](http://asia.ensembl.org/homo_sapiens/Gene/Summary?db=core;g=ENSG00000114391) | PDCL3P4 |
| JN675022.1 | 3q13.2 | chr3:113024277-113033435 | GTPBP8  NEPRO  NEPRO-AS1 | [CD200R1](http://asia.ensembl.org/homo_sapiens/Gene/Summary?db=core;g=ENSG00000163606) |  |
| JN675023.1 | 3q21.2 | chr3:125890459-125898836 | FAM86JP  ALG1L |  | LINC02614  ENPP7P4 |
| JN675025.1 | 3q27.2 | chr3:185562548-185571727 | LIPH  SENP2 |  |  |
| JN675026.1 | 4p16.1a | chr4:9121789-9131349 | FAM86kb  ALG1L14P | [FAM90A26](http://asia.ensembl.org/homo_sapiens/Gene/Summary?db=core;g=ENSG00000229924) | ENPP7P10 |
| JN675027.1 | 4p16.1b | chr4:9657964-9667026 | ENPP7P11  FAM86MP | [ALG1L3P](http://asia.ensembl.org/homo_sapiens/Gene/Summary?db=core;g=ENSG00000251087)  [Metazoa_SRP](http://asia.ensembl.org/homo_sapiens/Gene/Summary?db=core;g=ENSG00000203436) |  |
| JN675028.1 | 4p16.3a | chr4:241200-245565 | ZNF732 |  | ZNF876P |
| JN675029.1 | 4p16.3b | chr4:3977324-3986904 | ALG1L7P  FAM86EP  ENPP7P9 | [EVA1CP2](http://asia.ensembl.org/homo_sapiens/Gene/Summary?db=core;g=ENSG00000271977) |  |
| JN675030.1 | 4q13.2 | chr4:68597991-68603505 | UGT2B17 |  |  |
| JN675032.1 | 4q32.3 | chr4:164990383-165002916 | TRIM61  FAM218A  TRIM60 | [NACA3P](http://asia.ensembl.org/homo_sapiens/Gene/Summary?db=core;g=ENSG00000121089)  [TRIM75](http://asia.ensembl.org/homo_sapiens/Gene/Summary?db=core;g=ENSG00000250374) |  |
| JN675033.1 | 4q35.2 | chr4:190106259-190113546 | DUX4L7  DUX4L6  DUX4L5  DUX4L4  DUX4L1  DUX4L3  DUX4L2 | [DUX4L8](http://asia.ensembl.org/homo_sapiens/Gene/Summary?db=core;g=ENSG00000281720)  [DBET](http://asia.ensembl.org/homo_sapiens/Gene/Summary?db=core;g=ENSG00000281591)  [CLUHP4](http://asia.ensembl.org/homo_sapiens/Gene/Summary?db=core;g=ENSG00000249003) |  |
| JN675036.1 | 5q33.2 | chr5:154635953-154644654 |  | [MIR3141](http://asia.ensembl.org/homo_sapiens/Gene/Summary?db=core;g=ENSG00000264760)  [LARP1](http://asia.ensembl.org/homo_sapiens/Gene/Summary?db=core;g=ENSG00000155506)  [MIR1303](http://asia.ensembl.org/homo_sapiens/Gene/Summary?db=core;g=ENSG00000221552) |  |
| JN675037.1 | 5q33.3 | chr5:156657706-156666885 |  |  | SGCD |
| JN675038.1 | 6p11.2 | chr6:60654987-60660975 |  | [GAPDHP41](http://asia.ensembl.org/homo_sapiens/Gene/Summary?db=core;g=ENSG00000277172) |  |
| JN675039.1 | 6p21.1 | chr6:42893671-42903629 | BICRAL  RPL7L1  C6orf226  PTCRA  CNPY3 | [LINC02976](http://asia.ensembl.org/homo_sapiens/Gene/Summary?db=core;g=ENSG00000231113)  [LINC02976](http://asia.ensembl.org/homo_sapiens/Gene/Summary?db=core;g=ENSG00000231113)  [RPL24P4](http://asia.ensembl.org/homo_sapiens/Gene/Summary?db=core;g=ENSG00000181524)  [GNMT](http://asia.ensembl.org/homo_sapiens/Gene/Summary?db=core;g=ENSG00000124713) |  |
| JN675040.1 | 6p22.1 | chr6:28682590-28692958 | LINC00533 | [RPSAP2](http://asia.ensembl.org/homo_sapiens/Gene/Summary?db=core;g=ENSG00000237425) |  |
| JN675041.1 | 6q14.1 | chr6:77716945-77726366 |  |  | MEI4 |
| JN675042.1 | 6q25.1 | chr6:150859613-150862438 | PLEKHG1  PDCL3P5  MTHFD1L |  |  |
| JN675045.1 | 7q11.21 | chr7:66004702-66007397 | GUSB | [VKORC1L1](http://asia.ensembl.org/homo_sapiens/Gene/Summary?db=core;g=ENSG00000196715) |  |
| JN675046.1 | 7q22.2 | chr7:104748902-104752819 |  |  | LHFPL3  LHFPL3-AS1 |
| JN675047.1 | 7q34 | chr7:141752118-141756138 | WEE2-AS1  WEE2  RNU1-82P  TAS2R3  TAS2R4  TAS2R6P  TAS2R5 | [MTND1P3](http://asia.ensembl.org/homo_sapiens/Gene/Summary?db=core;g=ENSG00000239648)  [MYL6P4](http://asia.ensembl.org/homo_sapiens/Gene/Summary?db=core;g=ENSG00000240677)  [WEE2](http://asia.ensembl.org/homo_sapiens/Gene/Summary?db=core;g=ENSG00000214102)  [SSBP1](http://asia.ensembl.org/homo_sapiens/Gene/Summary?db=core;g=ENSG00000106028)  [MTCO1P55](http://asia.ensembl.org/homo_sapiens/Gene/Summary?db=core;g=ENSG00000241926)  [MTND2P5](http://asia.ensembl.org/homo_sapiens/Gene/Summary?db=core;g=ENSG00000224354) | SSBP1 |
| JN675048.1 | 8p22 | chr8:17907693-17916431 | PCM1 | [MTUS1-DT](http://asia.ensembl.org/homo_sapiens/Gene/Summary?db=core;g=ENSG00000253944) | FGL1 |
| JN675049.1 | 8p23.1a | chr8:7497875-7507337 | SPAG11B  DEFB104B  DEFB106B  DEFB105B  PRR23D1 | [FAM90A6P](http://asia.ensembl.org/homo_sapiens/Gene/Summary?db=core;g=ENSG00000248944)  [FAM90A7](http://asia.ensembl.org/homo_sapiens/Gene/Summary?db=core;g=ENSG00000285975)  [FAM90A21P](http://asia.ensembl.org/homo_sapiens/Gene/Summary?db=core;g=ENSG00000234749) | DEFB107B |
| JN675050.1 | 8p23.1b | chr8:8197178-8206699 | FAM85B  FAM86B3P  ALG1L13P |  | ENPP7P1 |
| JN675051.1 | 8p23.1c | chr8:12216461-12225988 | ALG1L11P | [FAM66D](http://asia.ensembl.org/homo_sapiens/Gene/Summary?db=core;g=ENSG00000255052) |  |
|  |  |  | FAM86B1 | [FAM90A2P](http://asia.ensembl.org/homo_sapiens/Gene/Summary?db=core;g=ENSG00000205879) |  |
|  |  |  | ENPP7P12 | [ALG1L11P](http://asia.ensembl.org/homo_sapiens/Gene/Summary?db=core;g=ENSG00000249889) |  |
| JN675052.1 | 8p23.1d | chr8:12458983-12468498 | ALG1L12P  FAM86B2 | [DEFB109A](http://asia.ensembl.org/homo_sapiens/Gene/Summary?db=core;g=ENSG00000242296)  [FAM90A25P](http://asia.ensembl.org/homo_sapiens/Gene/Summary?db=core;g=ENSG00000251402) | ENPP7P6 |
| JN675055.1 | 8q24.3b | chr8:145021262-145028825 | ZNF252P  TMED10P1  ZNF252P-AS1  C8orf33 |  |  |
| JN675056.1 | 9q34.3 | chr9:136780314-136789776 | LCN6  MIR6722  LCN8  LCN15  ATP6V1G1P3  TMEM141  CCDC183  CCDC183-AS1  RABL6  NCLP1 | [DIPK1B](http://asia.ensembl.org/homo_sapiens/Gene/Summary?db=core;g=ENSG00000165716)  [SNORA17B](http://asia.ensembl.org/homo_sapiens/Gene/Summary?db=core;g=ENSG00000280496)  [SNORA17A](http://asia.ensembl.org/homo_sapiens/Gene/Summary?db=core;g=ENSG00000274998)  [LCN10](http://asia.ensembl.org/homo_sapiens/Gene/Summary?db=core;g=ENSG00000187922)  [MIR4292](http://asia.ensembl.org/homo_sapiens/Gene/Summary?db=core;g=ENSG00000265806)  [PHPT1](http://asia.ensembl.org/homo_sapiens/Gene/Summary?db=core;g=ENSG00000054148)  [AJM1](http://asia.ensembl.org/homo_sapiens/Gene/Summary?db=core;g=ENSG00000232434)  [SNHG7](http://asia.ensembl.org/homo_sapiens/Gene/Summary?db=core;g=ENSG00000233016) |  |
| JN675057.1 | 9q34.11 | chr9:128850236-128857457 | ENDOG  SPOUT1  LRRC8A | [TBC1D13](http://asia.ensembl.org/homo_sapiens/Gene/Summary?db=core;g=ENSG00000107021) | KYAT1 |
| JN675058.1 | 10p12.1 | chr10:26893470-26894437 | ABI1 | [FAM238C](http://asia.ensembl.org/homo_sapiens/Gene/Summary?db=core;g=ENSG00000290706) | CKS1BP2  ZNF33BP1 |
| JN675059.1 | 10p14 | chr10:6824179-6833641 |  | [LINC00706](http://asia.ensembl.org/homo_sapiens/Gene/Summary?db=core;g=ENSG00000281186) | LINC00707 |
| JN675060.1 | 10q24.2 | chr10:99820812-99827959 | NANOGP6 |  | ABCC2 |
| JN675061.1 | 11p15.4 | chr11:3447426-3456979 | FAM86GP | [OR7E12P](http://asia.ensembl.org/homo_sapiens/Gene/Summary?db=core;g=ENSG00000189398)  [TSSC2](http://asia.ensembl.org/homo_sapiens/Gene/Summary?db=core;g=ENSG00000223756) | ENPP7P15 |
| JN675062.1 | 11q12.1 | chr11:58999975-59005723 | LINC01358  PHBP3  GLYATL1P1 | [GLYATL1P4](http://asia.ensembl.org/homo_sapiens/Gene/Summary?db=core;g=ENSG00000254399) | GLYATL1 |
| JN675063.1 | 11q12.3 | chr11:62368491-62383091 | SCGB1A1 | [NPM1P35](http://asia.ensembl.org/homo_sapiens/Gene/Summary?db=core;g=ENSG00000255213)  [AHNAK](http://asia.ensembl.org/homo_sapiens/Gene/Summary?db=core;g=ENSG00000124942) | ASRGL1  RCC2P6 |
| JN675064.1 | 11q22.1 | chr11:101695063-101704528 | LINC01709  RNU6-965P |  | TRPC6 |
| JN675065.1 | 11q23.3 | chr11:118721015-118730174 | TREHP1  RNU6-376P  DDX6 | [TREH](http://asia.ensembl.org/homo_sapiens/Gene/Summary?db=core;g=ENSG00000118094) |  |
| JN675067.1 | 12q13.2 | chr12:55333431-55334399 | OR6C5P  OR6C1  OR6C3  OR6C7P  OR6C75 | [OR6C72P](http://asia.ensembl.org/homo_sapiens/Gene/Summary?db=core;g=ENSG00000205331)  [OR6C6](http://asia.ensembl.org/homo_sapiens/Gene/Summary?db=core;g=ENSG00000188324)  [OR6C71P](http://asia.ensembl.org/homo_sapiens/Gene/Summary?db=core;g=ENSG00000203408)  [OR6C66P](http://asia.ensembl.org/homo_sapiens/Gene/Summary?db=core;g=ENSG00000233606) |  |
| JN675068.1 | 12q14.1 | chr12:58327459-58336915 |  |  |  |
| JN675069.1 | 12q24.11 | chr12:110570038-110571520 |  | [RAD9B](http://asia.ensembl.org/homo_sapiens/Gene/Summary?db=core;g=ENSG00000151164)  [TCTN1](http://asia.ensembl.org/homo_sapiens/Gene/Summary?db=core;g=ENSG00000204852)  [RN7SL387P](http://asia.ensembl.org/homo_sapiens/Gene/Summary?db=core;g=ENSG00000263537)  [HVCN1](http://asia.ensembl.org/homo_sapiens/Gene/Summary?db=core;g=ENSG00000122986) | PPTC7 |
| JN675070.1 | 12q24.33 | chr12:133090536-133096478 | ZNF84  ZNF891  RPL23AP67  ZNF10 | [ZNF26](http://asia.ensembl.org/homo_sapiens/Gene/Summary?db=core;g=ENSG00000198393)  [PTP4A1P2](http://asia.ensembl.org/homo_sapiens/Gene/Summary?db=core;g=ENSG00000255807) | ZNF140 |
| JN675071.1 | 14q11.2 | chr14:24009696-24015776 | DHRS4-AS1  DHRS4L2  DHRS4L1 | [CARMIL3](http://asia.ensembl.org/homo_sapiens/Gene/Summary?db=core;g=ENSG00000186648)  [CPNE6](http://asia.ensembl.org/homo_sapiens/Gene/Summary?db=core;g=ENSG00000100884)  [DHRS4](http://asia.ensembl.org/homo_sapiens/Gene/Summary?db=core;g=ENSG00000157326) |  |
| JN675072.1 | 14q32.33 | chr14:105673313-105676203 | IGHG2  MIR8071-2  COPDA1  IGHGP  ELK2AP  IGHA1 | [IGHEP1](http://asia.ensembl.org/homo_sapiens/Gene/Summary?db=core;g=ENSG00000253692)  [IGHG4](http://asia.ensembl.org/homo_sapiens/Gene/Summary?db=core;g=ENSG00000211892)  [MIR8071-1](http://asia.ensembl.org/homo_sapiens/Gene/Summary?db=core;g=ENSG00000274172)  [MIR8071-2](http://asia.ensembl.org/homo_sapiens/Gene/Summary?db=core;g=ENSG00000277030) |  |
| JN675073.1 | 15q25.2 | chr15:84160268-84163612 | EFL1P1  DNM1P41  UBE2Q2L  CSPG4P11 | [UBE2Q2P16](http://asia.ensembl.org/homo_sapiens/Gene/Summary?db=core;g=ENSG00000259511) |  |
| JN675074.1 | 16p11.2 | chr16:34997026-34999771 | LINC02184 | [CCNYL3](http://asia.ensembl.org/homo_sapiens/Gene/Summary?db=core;g=ENSG00000216671) |  |
| JN675089.1 | 16p13.3 | chr16:2926159-2927660 | FLYWCH2  RPL23AP86 | [KREMEN2](http://asia.ensembl.org/homo_sapiens/Gene/Summary?db=core;g=ENSG00000131650)  [PKMYT1](http://asia.ensembl.org/homo_sapiens/Gene/Summary?db=core;g=ENSG00000127564)  [PAQR4](http://asia.ensembl.org/homo_sapiens/Gene/Summary?db=core;g=ENSG00000162073) | FLYWCH1 |
| JN675075.1 | 17p13.1 | chr17:8056337-8063901 | ALOX15B | [GUCY2D](http://asia.ensembl.org/homo_sapiens/Gene/Summary?db=core;g=ENSG00000132518) |  |
|  |  |  | ALOXE3P1 | [HES7](http://asia.ensembl.org/homo_sapiens/Gene/Summary?db=core;g=ENSG00000179111) |  |
|  |  |  | ALOX12B | [GOLGA2P7](http://asia.ensembl.org/homo_sapiens/Gene/Summary?db=core;g=ENSG00000225151) |  |
|  |  |  | MIR4314 | [RN7SL331P](http://asia.ensembl.org/homo_sapiens/Gene/Summary?db=core;g=ENSG00000278422) |  |
|  |  |  | ALOXE3 |  |  |
| JN675076.1 | 19p12a | chr19:20276591-20286703 | BNIP3P18 | [BNIP3P17](http://asia.ensembl.org/homo_sapiens/Gene/Summary?db=core;g=ENSG00000271524)  [BNIP3P19](http://asia.ensembl.org/homo_sapiens/Gene/Summary?db=core;g=ENSG00000271638) |  |
| JN675077.1 | 19p12b | chr19:27637986-27646483 | LINC00662 |  | ERVK-28 |
| JN675078.1 | 19p12c | chr19:22575022-22581759 | RNU6-1179P  GOLGA2P9  RN7SL860P  RAD54L2P1  LINC01785 | [ZNF98](http://asia.ensembl.org/homo_sapiens/Gene/Summary?db=core;g=ENSG00000197360)  [LINC01233](http://asia.ensembl.org/homo_sapiens/Gene/Summary?db=core;g=ENSG00000269364)  [ZNF492](http://asia.ensembl.org/homo_sapiens/Gene/Summary?db=core;g=ENSG00000229676) |  |
| JN675079.1 | 19p13.3 | chr19:385095-387637 | THEG  C2CD4C  SHC2 | [MIER2](http://asia.ensembl.org/homo_sapiens/Gene/Summary?db=core;g=ENSG00000105556) |  |
| JN675080.1 | 19q11 | chr19:27637590-27646453 | LINC00662 |  | ERVK-28 |
| JN675081.1 | 19q13.12a | chr19:35572305-35576532 | GAPDHS  TMEM147-AS1  TMEM147  ATP4A  PMIS2  LINC01766  HAUS5-DT | [DMKN](http://asia.ensembl.org/homo_sapiens/Gene/Summary?db=core;g=ENSG00000161249)  [SBSN](http://asia.ensembl.org/homo_sapiens/Gene/Summary?db=core;g=ENSG00000189001)  [HAUS5](http://asia.ensembl.org/homo_sapiens/Gene/Summary?db=core;g=ENSG00000249115)  [RBM42](http://asia.ensembl.org/homo_sapiens/Gene/Summary?db=core;g=ENSG00000126254) |  |
| JN675082.1 | 19q13.12b | chr19:37106647-37116164 | ZNF585A |  | ZNF420 |
| JN675083.1 | 19q13.41 | chr19:52742028-52750454 | ZNF611 | [ZNF83](http://asia.ensembl.org/homo_sapiens/Gene/Summary?db=core;g=ENSG00000167766)  [ZNF28](http://asia.ensembl.org/homo_sapiens/Gene/Summary?db=core;g=ENSG00000198538)  [PABPN1P2](http://asia.ensembl.org/homo_sapiens/Gene/Summary?db=core;g=ENSG00000255875) | ZNF600 |
| JN675084.1 | 19q13.42 | chr19:53359095-53364791 | ZNF845  ZNF525  ZNF765 | [FAM90A28P](http://asia.ensembl.org/homo_sapiens/Gene/Summary?db=core;g=ENSG00000269118)  [VN1R6P](http://asia.ensembl.org/homo_sapiens/Gene/Summary?db=core;g=ENSG00000174677)  [RPL39P36](http://asia.ensembl.org/homo_sapiens/Gene/Summary?db=core;g=ENSG00000239912) |  |
| JN675085.1 | 20q11.22 | chr20:34127754-34136578 | RALY  EIF2S2  RPS2P1 | [ASIP](http://asia.ensembl.org/homo_sapiens/Gene/Summary?db=core;g=ENSG00000101440) |  |
| JN675086.1 | 21q21.1 | chr21:18561341-18569644 | MIR548XHG |  |  |
| JN675087.1 | 22q11.21 | chr22:18938674-18947848 | DGCR6  PRODH  DGCR5 | [FAM230F](http://asia.ensembl.org/homo_sapiens/Gene/Summary?db=core;g=ENSG00000276095)  [CELSR1P1](http://asia.ensembl.org/homo_sapiens/Gene/Summary?db=core;g=ENSG00000237517) |  |
| JN675088.1 | 22q11.23 | chr22:23537740-23546900 | LINC02557  IGLL1 | [FAM230I](http://asia.ensembl.org/homo_sapiens/Gene/Summary?db=core;g=ENSG00000178248) | PCAT14 |
| JN675090.1 | Xq11.1 | chrX:62740079-62742584 |  | [SSBL2P](http://asia.ensembl.org/homo_sapiens/Gene/Summary?db=core;g=ENSG00000236852) |  |
| JN675091.1 | Xq12 | chrX:66464290-66466342 | PKMP2 |  |  |
| JN675092.1 | Xq28a | chrX:154613097-154615762 | ATF4P2  ATF4P1  FAM223B  IKBKGP1  CTAG1A  CTAG1B | [IKBKG](http://asia.ensembl.org/homo_sapiens/Gene/Summary?db=core;g=ENSG00000269335)  [FAM223A](http://asia.ensembl.org/homo_sapiens/Gene/Summary?db=core;g=ENSG00000279245)  [CTAG2](http://asia.ensembl.org/homo_sapiens/Gene/Summary?db=core;g=ENSG00000126890)  [OR3B1P](http://asia.ensembl.org/homo_sapiens/Gene/Summary?db=core;g=ENSG00000237619)  [GAB3](http://asia.ensembl.org/homo_sapiens/Gene/Summary?db=core;g=ENSG00000160219) |  |
| JN675093.1 | Xq28b | chrX:154608423-154615762 |  | [IKBKG](http://asia.ensembl.org/homo_sapiens/Gene/Summary?db=core;g=ENSG00000269335)  [FAM223A](http://asia.ensembl.org/homo_sapiens/Gene/Summary?db=core;g=ENSG00000279245)  [ATF4P2](http://asia.ensembl.org/homo_sapiens/Gene/Summary?db=core;g=ENSG00000273041)  [CTAG1A](http://asia.ensembl.org/homo_sapiens/Gene/Summary?db=core;g=ENSG00000268651)  [CTAG1B](http://asia.ensembl.org/homo_sapiens/Gene/Summary?db=core;g=ENSG00000184033)  [ATF4P1](http://asia.ensembl.org/homo_sapiens/Gene/Summary?db=core;g=ENSG00000213338)  [FAM223B](http://asia.ensembl.org/homo_sapiens/Gene/Summary?db=core;g=ENSG00000272681)  [IKBKGP1](http://asia.ensembl.org/homo_sapiens/Gene/Summary?db=core;g=ENSG00000275882)  [CTAG2](http://asia.ensembl.org/homo_sapiens/Gene/Summary?db=core;g=ENSG00000126890)  [OR3B1P](http://asia.ensembl.org/homo_sapiens/Gene/Summary?db=core;g=ENSG00000237619)  [GAB3](http://asia.ensembl.org/homo_sapiens/Gene/Summary?db=core;g=ENSG00000160219) |  |
| JN675094.1 | Yp11.2 | chrY:6958400-6965343 |  | [AMELY](http://asia.ensembl.org/homo_sapiens/Gene/Summary?db=core;g=ENSG00000099721)  [ATP5PFP1](http://asia.ensembl.org/homo_sapiens/Gene/Summary?db=core;g=ENSG00000237701) | TBL1Y |
| JN675095.1 | Yq11.23a | chrY:24251690-24254888 | UBE2Q2P4Y  LINC00265-2P  CICP2  LINC00266-2P | [CSPG4P3Y](http://asia.ensembl.org/homo_sapiens/Gene/Summary?db=core;g=ENSG00000172342)  [RN7SL818P](http://asia.ensembl.org/homo_sapiens/Gene/Summary?db=core;g=ENSG00000274234)  [SEPTIN14P22](http://asia.ensembl.org/homo_sapiens/Gene/Summary?db=core;g=ENSG00000248792)  [GOLGA2P2Y](http://asia.ensembl.org/homo_sapiens/Gene/Summary?db=core;g=ENSG00000239533) |  |
| JN675096.1 | Yq11.23b | chrY:25415255-25418454 |  | [SEPTIN14P23](http://asia.ensembl.org/homo_sapiens/Gene/Summary?db=core;g=ENSG00000228786)  [CICP1](http://asia.ensembl.org/homo_sapiens/Gene/Summary?db=core;g=ENSG00000233652)  [LINC00265-3P](http://asia.ensembl.org/homo_sapiens/Gene/Summary?db=core;g=ENSG00000233944)  [UBE2Q2P5Y](http://asia.ensembl.org/homo_sapiens/Gene/Summary?db=core;g=ENSG00000223636)  [GOLGA2P3Y](http://asia.ensembl.org/homo_sapiens/Gene/Summary?db=core;g=ENSG00000172297)  [RN7SL725P](http://asia.ensembl.org/homo_sapiens/Gene/Summary?db=core;g=ENSG00000278242)  [CSPG4P4Y](http://asia.ensembl.org/homo_sapiens/Gene/Summary?db=core;g=ENSG00000172294) |  |
