# Supplementary material for: HERVK-mediated regulation of neighboring genes: implications for breast cancer prognosis
Source: Retrovirology. 2024 Feb 22;21:4. doi: 10.1186/s12977-024-00636-z (PMC10885364; doi:10.1186/s12977-024-00636-z)
Supplement: Supplementary file 5 — Additional file 5: Figure S1. Correlation of HERV-K provirus expression with neighboring genomes in various GSE dataset samples. [file 12977_2024_636_MOESM5_ESM.pdf]

Expression correlation of abnormally expressed HERV-K provirus and its neighborhood genomes in different samples of the GSE dataset. When HERV-K expression was significantly correlated with its neighboring genes, the corresponding correlation coefficients (P< 0.05). White indicates no correlation, blue indicates positive correlation, and red indicates negative correlation.

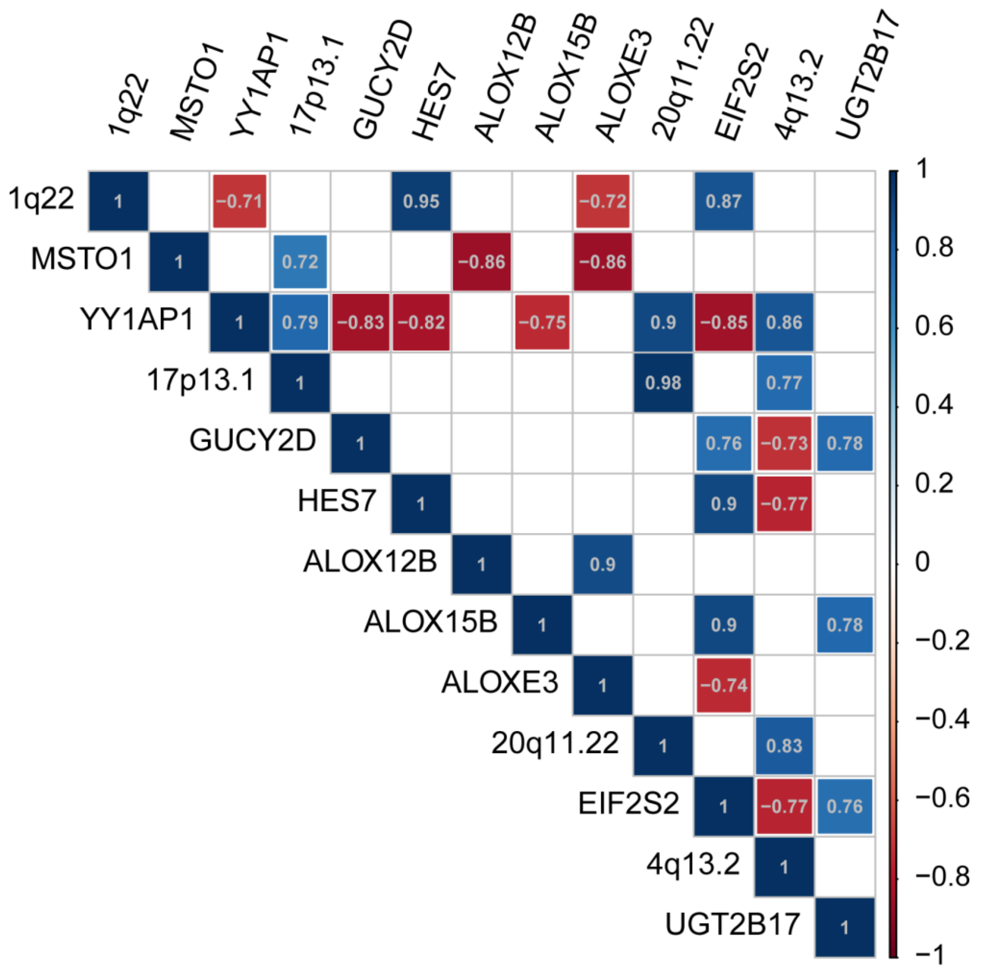

Figure 1 GSE96860 data set correlation heat map (76NF2V cells as normal control)

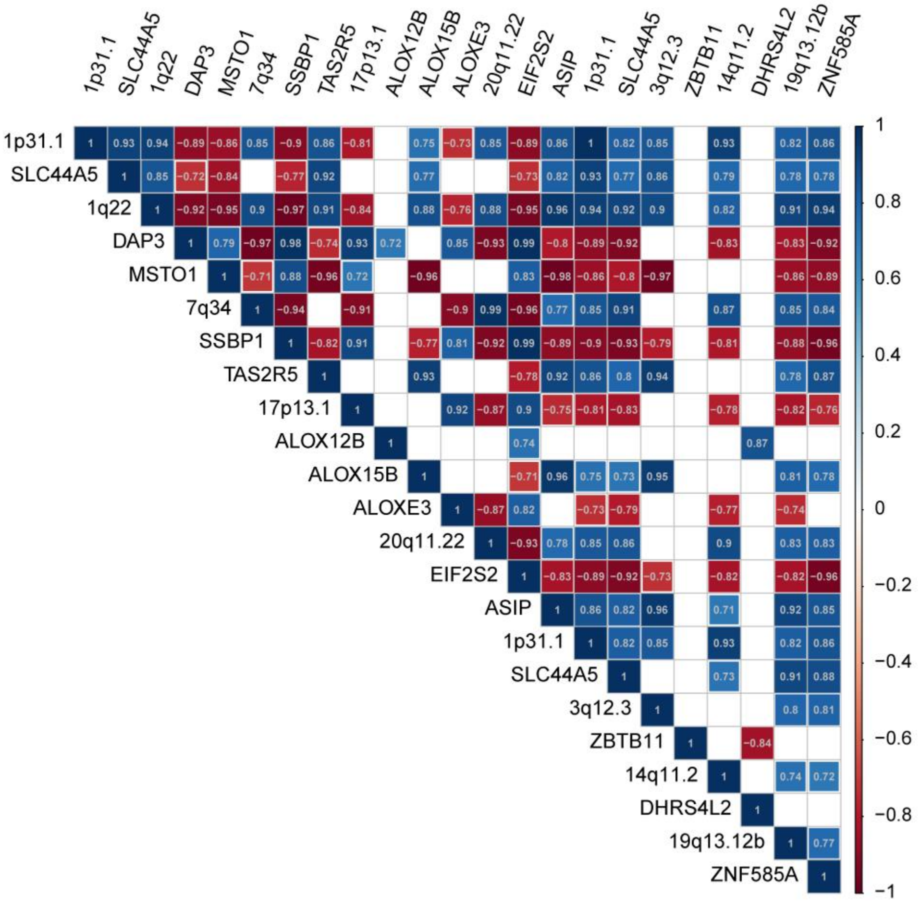

Figure 2 GSE96860 data set correlation heat map (MCF10A cells as normal control)

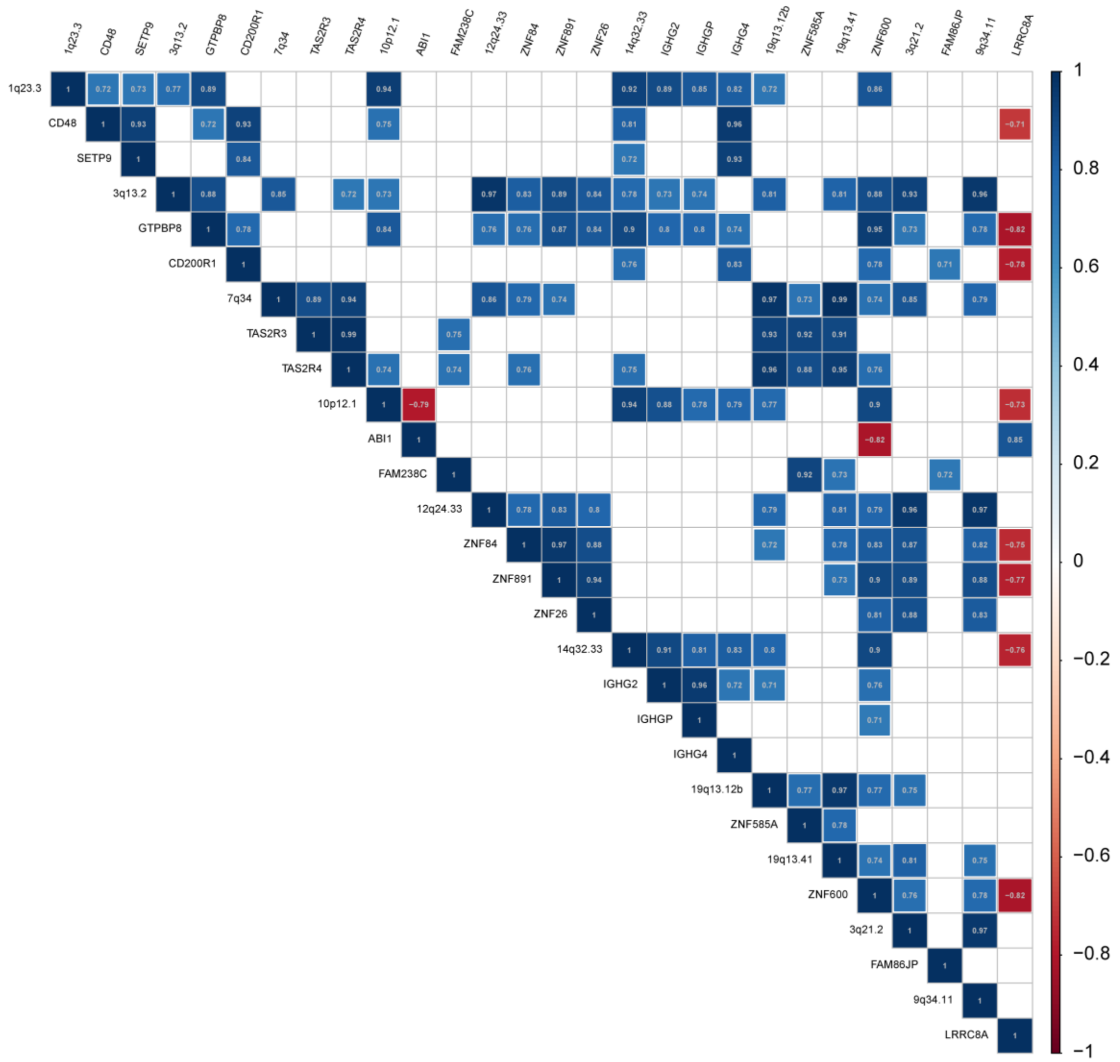

Figure 3 GSE52194 data set HER2+ breast cancer sample correlation heat map

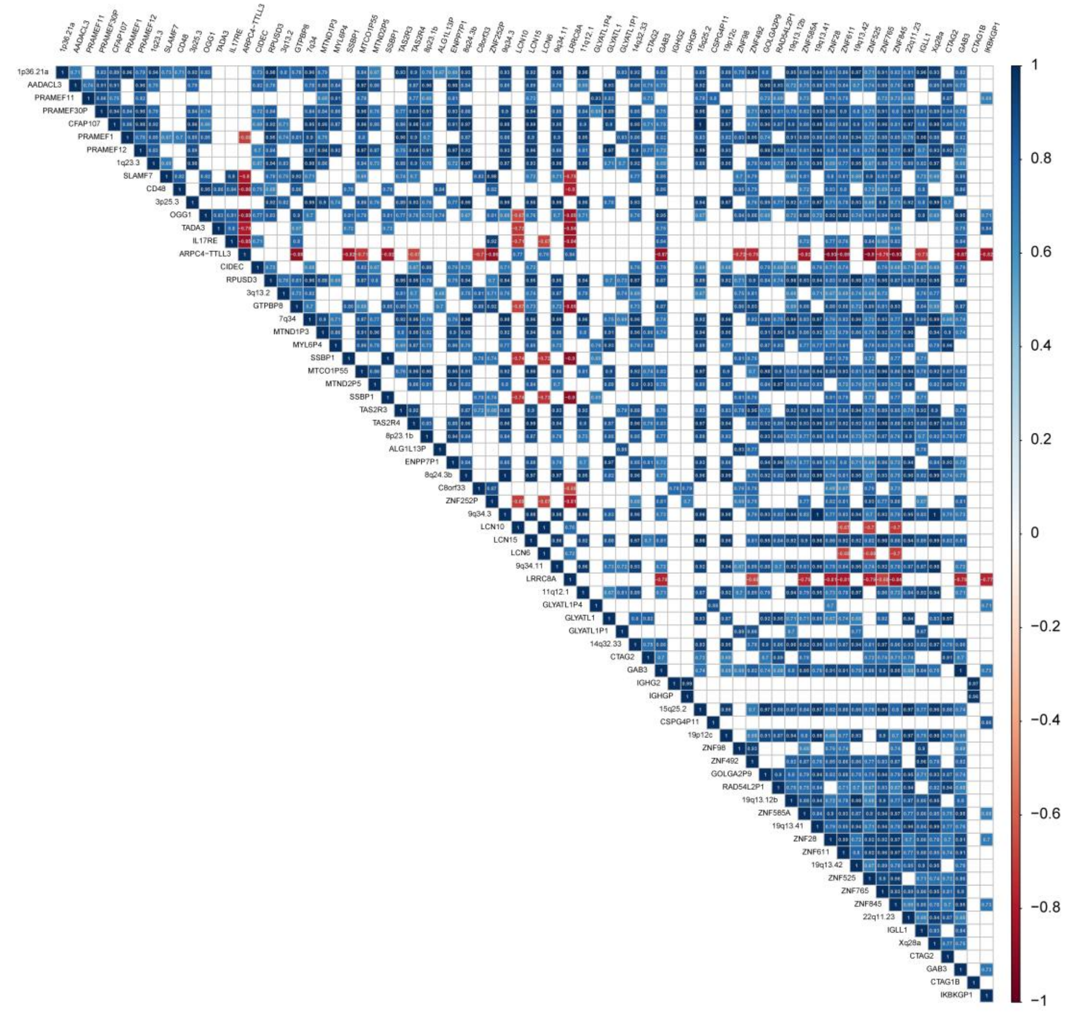

Figure 4 Heat map related to TNBC breast cancer samples in GSE52194 dataset

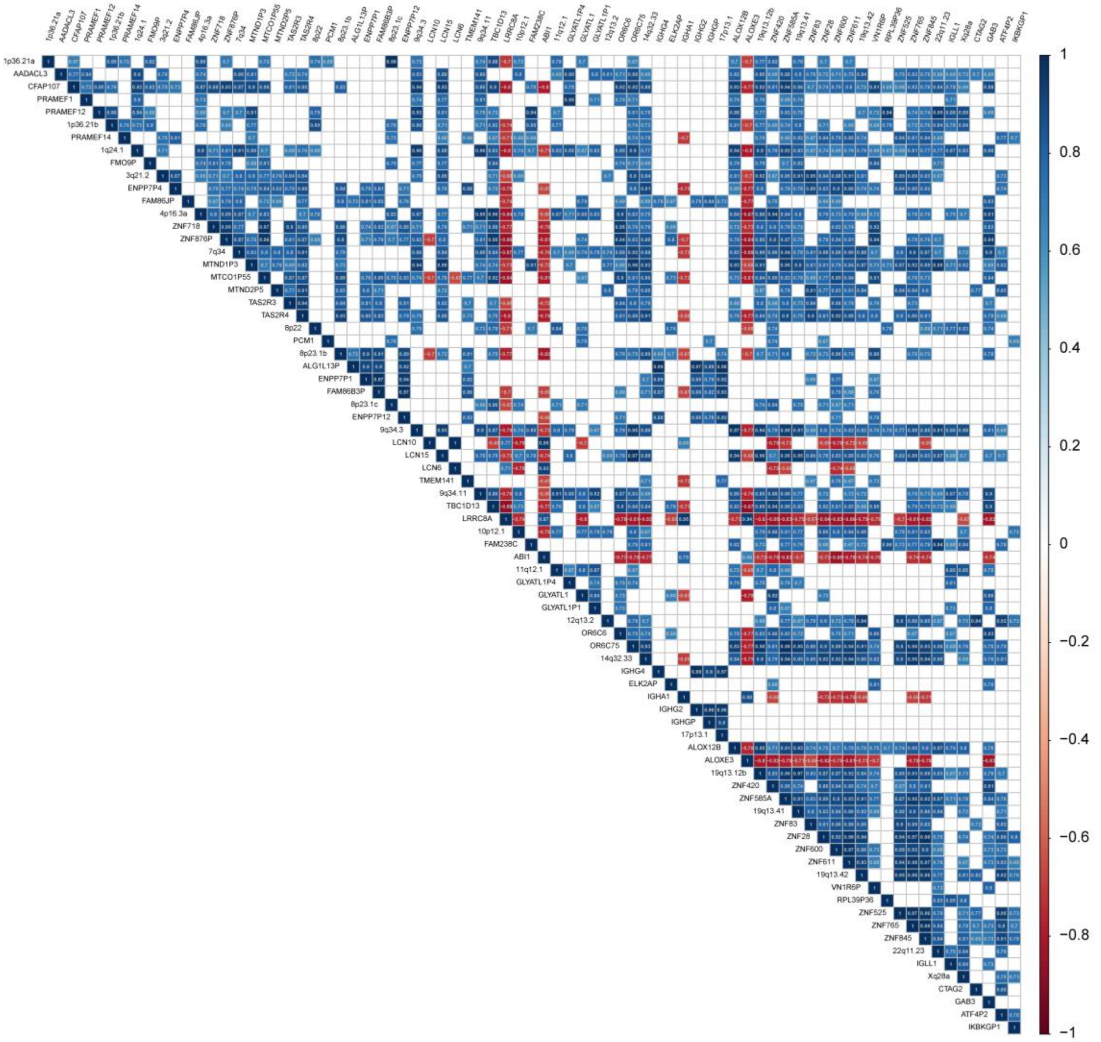

Figure 5 Heat map of non-TNBC breast cancer samples from GSE52194 dataset

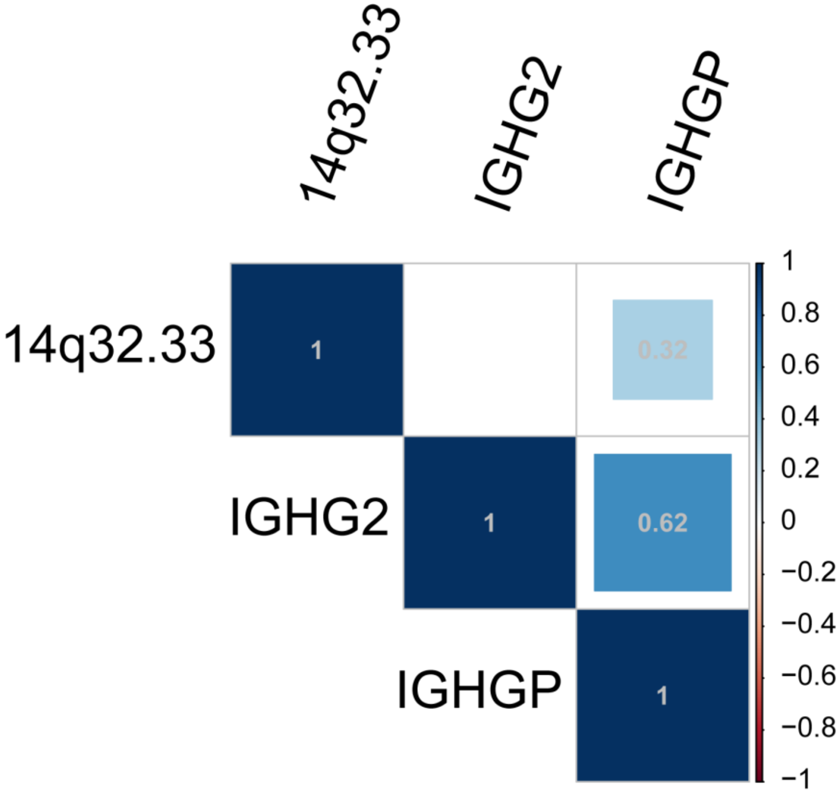

Figure 6 GSE103001 data set ER+ breast cancer sample correlation heat map

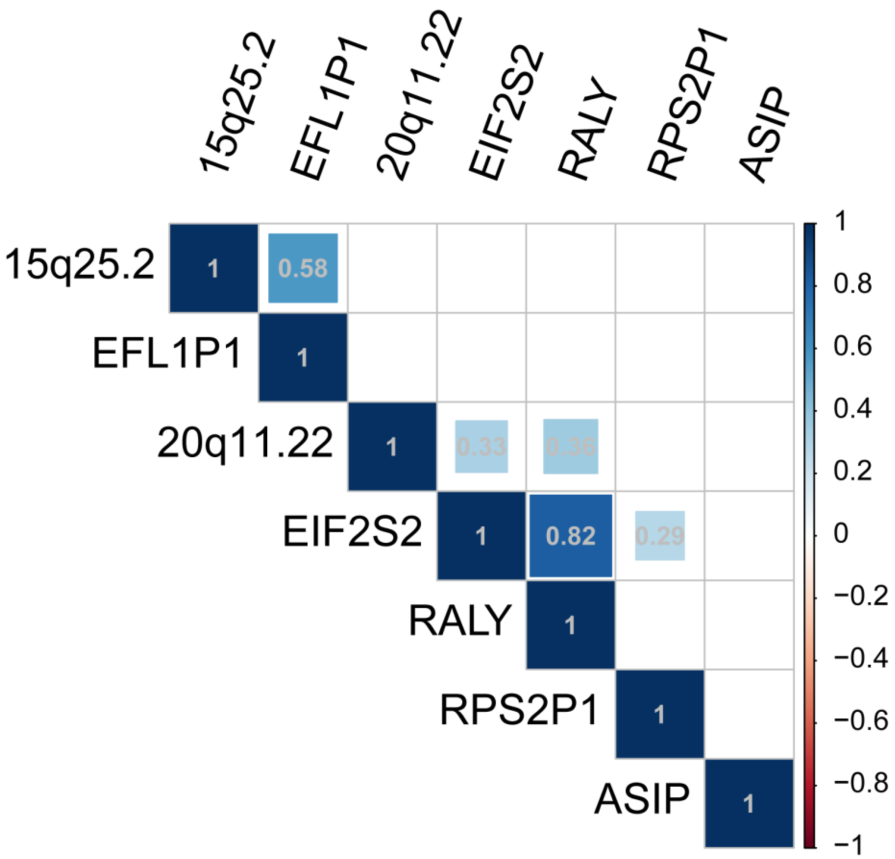

Figure 7 GSE58135 data set TNBC breast cancer sample correlation heat map

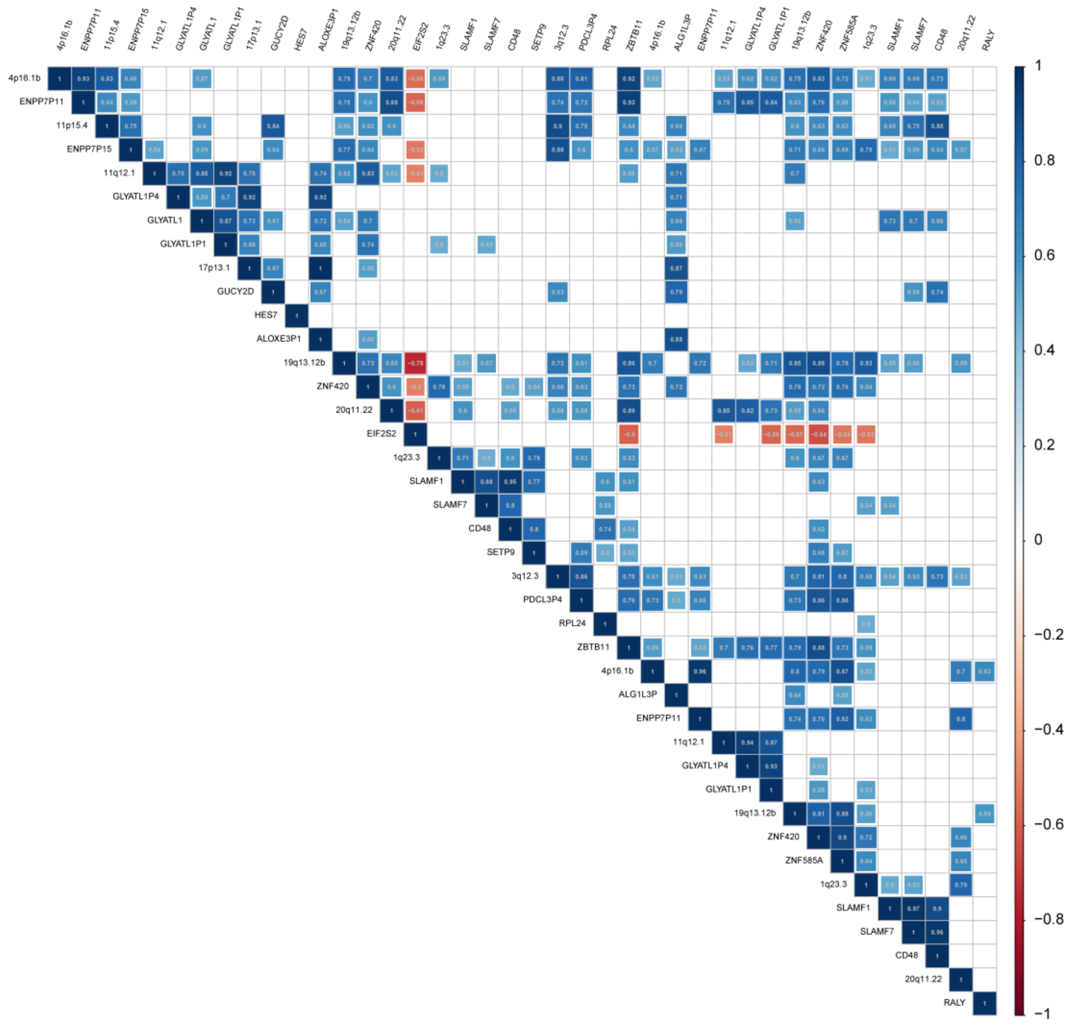

Figure 8 GSE45419 data set correlation heat map (ER+, HER2+, TNBC)

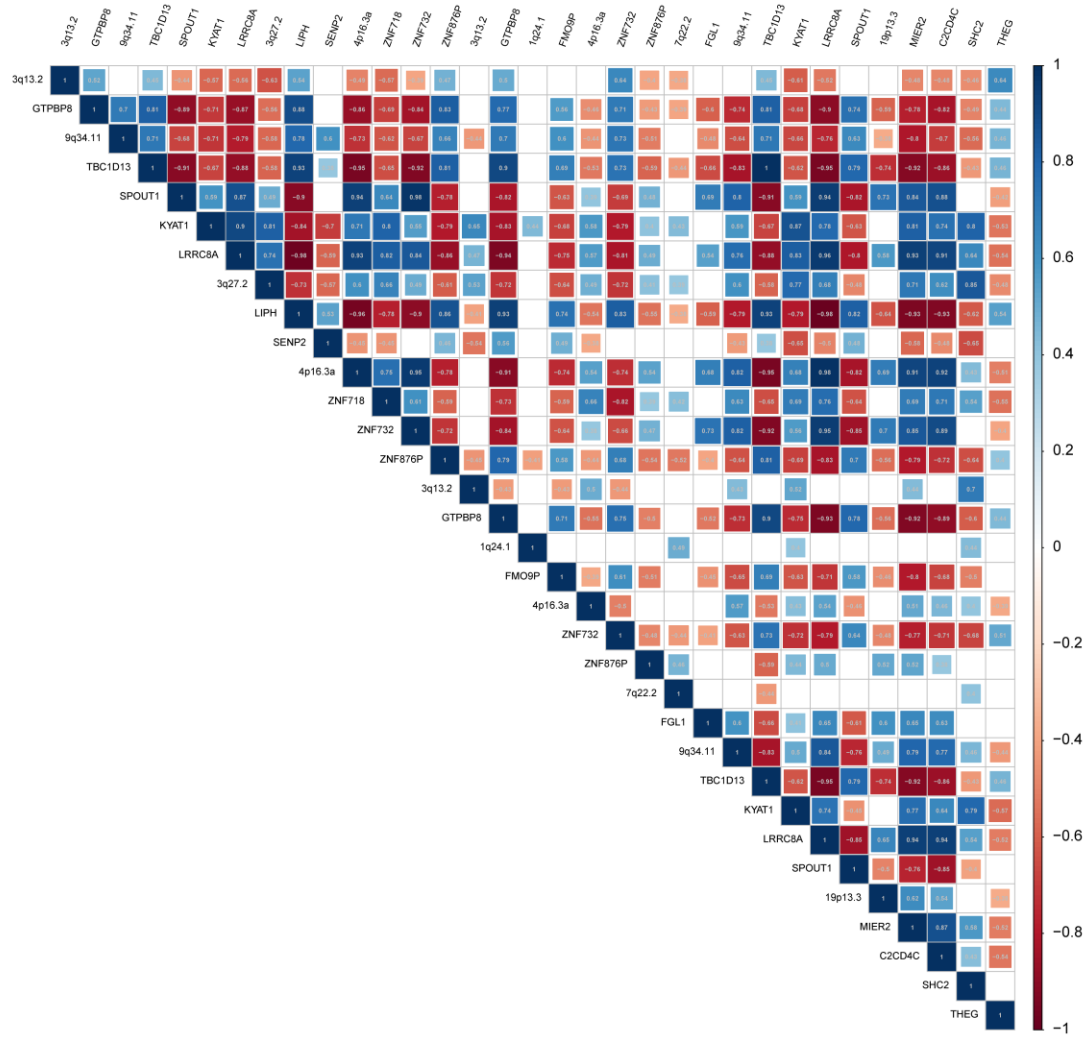

Figure 9 GSE171957 data set correlation heat map (MDA-MB-231 and HCC1937 cell line)
