# Supplementary figures and images for: HERVK-mediated regulation of neighboring genes: implications for breast cancer prognosis
Source: Retrovirology. 2024 Feb 22;21:4. doi: 10.1186/s12977-024-00636-z (PMC10885364; doi:10.1186/s12977-024-00636-z)

MCF-7

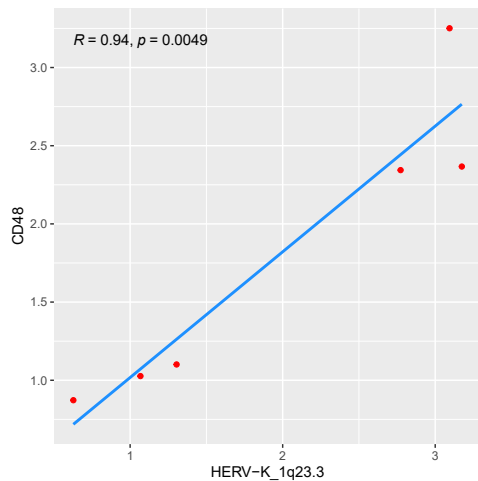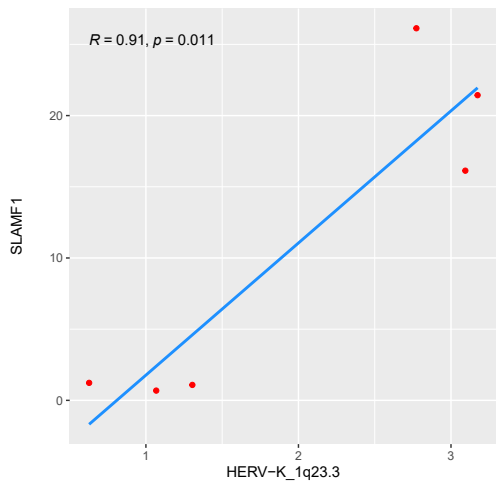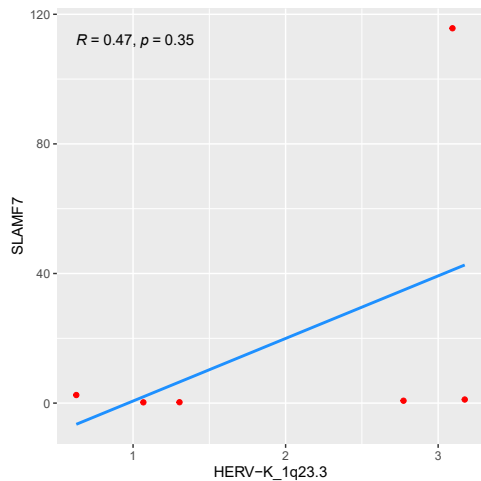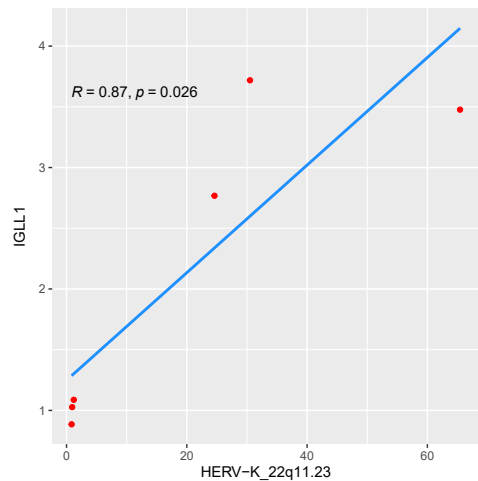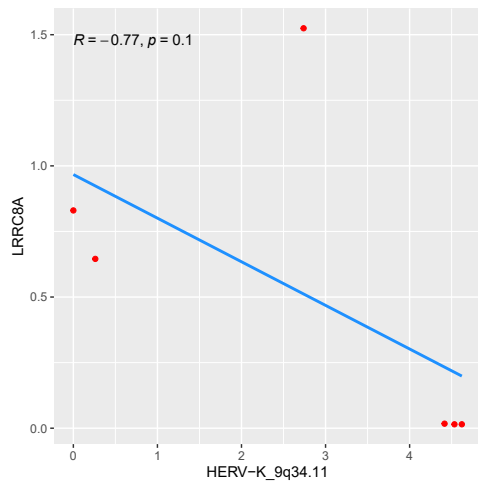

AU565

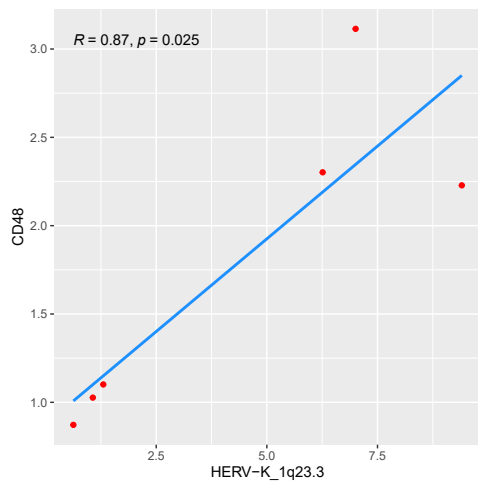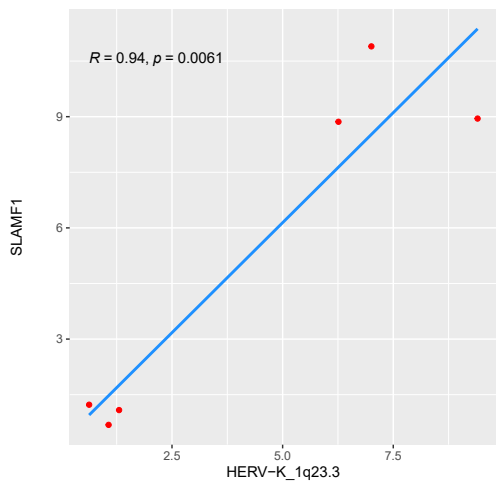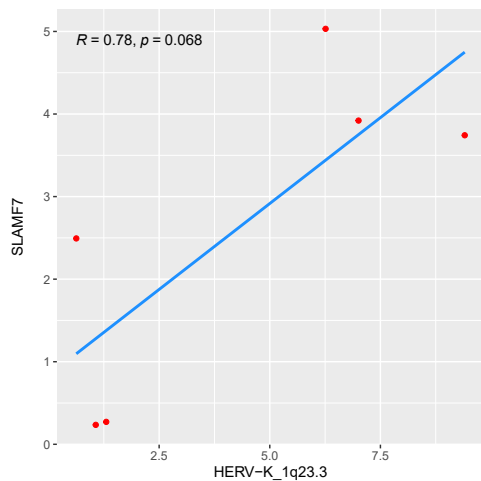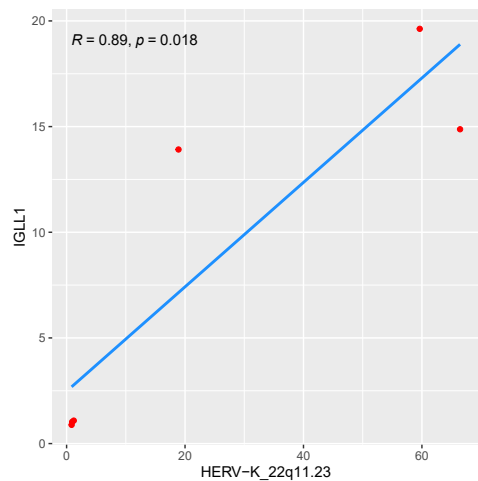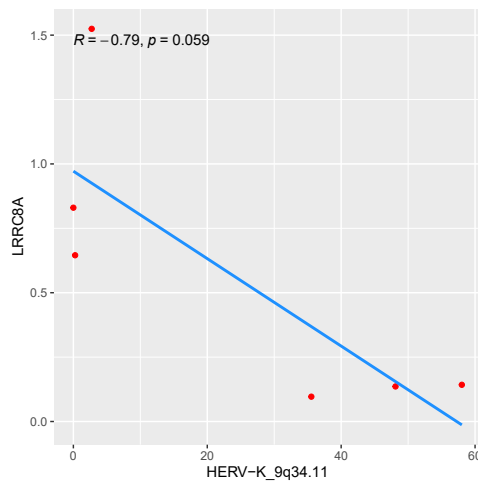

MB468

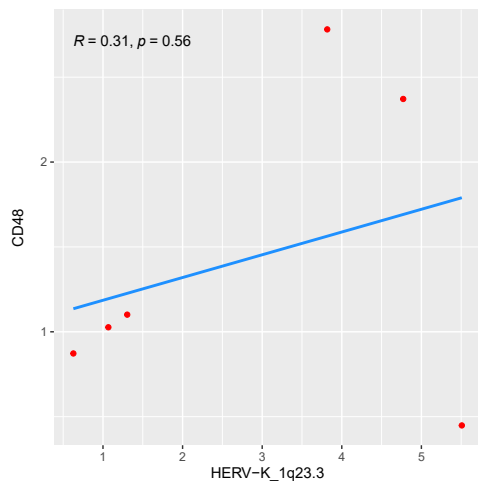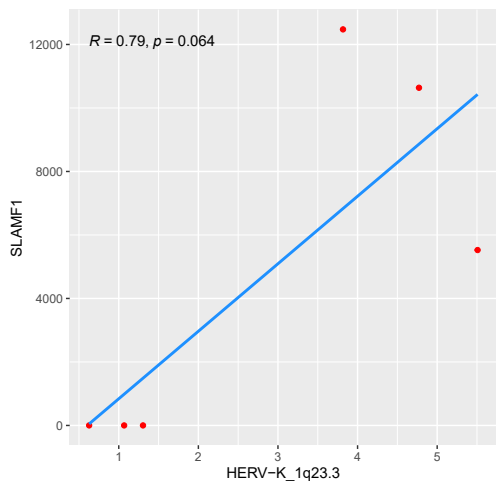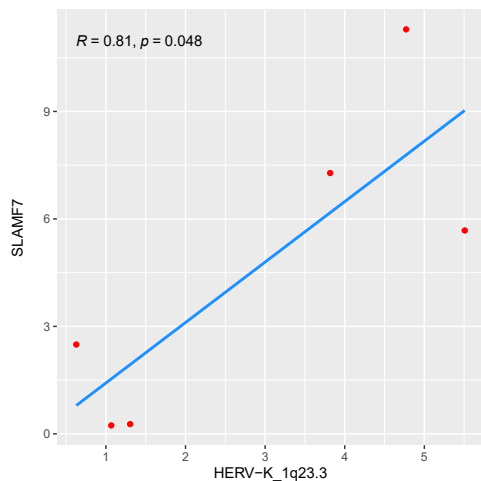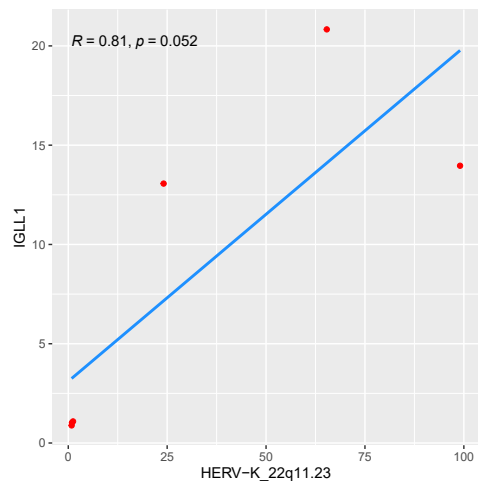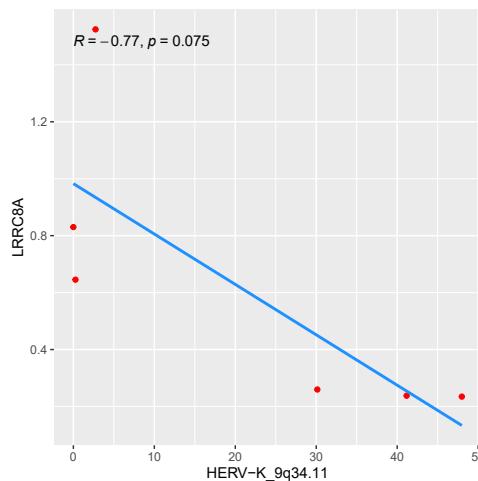

MDA-MB-231

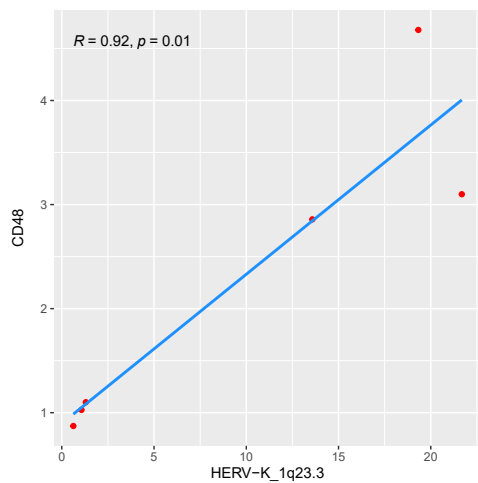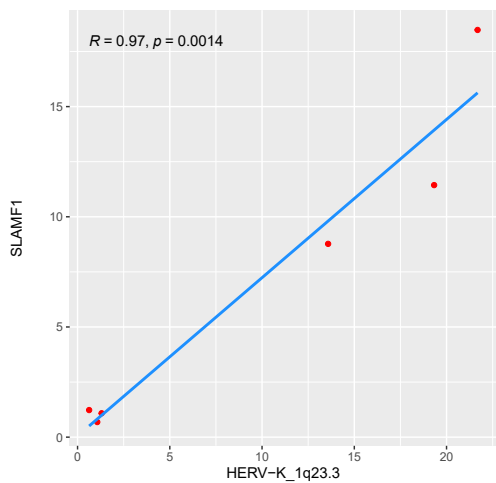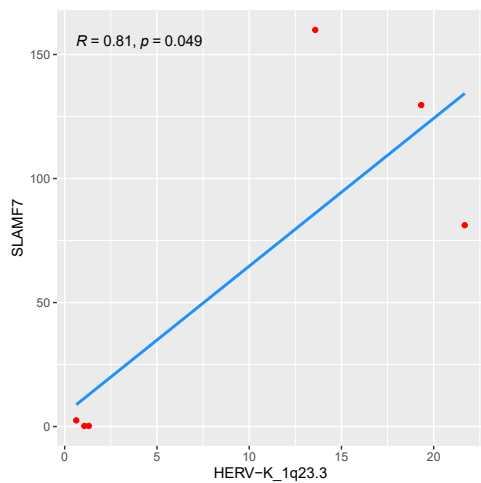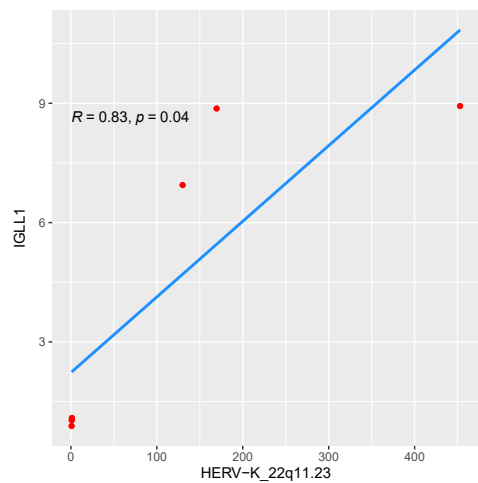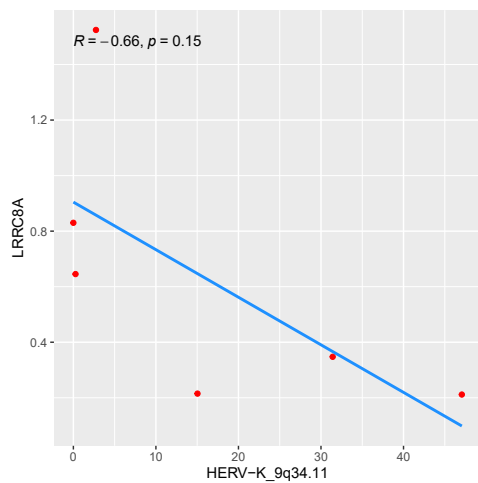

Supplement: Supplementary file 6 — Additional file 6: Figure S2. Correlation analysis of HERV-K with neighboring genes. [file 12977_2024_636_MOESM6_ESM.pdf]
